# Comparative proteomic analysis of exosomes derived from endothelial cells and Schwann cells

**DOI:** 10.1371/journal.pone.0290155

**Published:** 2023-08-18

**Authors:** Lei Wang, XueRong Lu, Michael Chopp, Chao Li, Yi Zhang, Alexandra Szalad, Xian Shuang Liu, Zheng Gang Zhang

**Affiliations:** 1 Department of Neurology, Henry Ford Health, Detroit, Michigan, United States of America; 2 Department of Physics, Oakland University, Rochester, Michigan, United States of America; Normandie Universite, UNITED STATES

## Abstract

Exosomes derived from endothelial cells and Schwann cells have been employed as novel treatments of neurological diseases, including peripheral neuropathy. Exosomal cargo plays a critical role in mediating recipient cell function. In this study, we thus performed a comprehensive proteomic analysis of exosomes derived from healthy mouse dermal microvascular endothelial cells (EC-Exo) and healthy mouse Schwann cells (SC-Exo). We detected 1,817and 1,579 proteins in EC-Exo and SC-Exo, respectively. Among them, 1506 proteins were present in both EC-Exo and SC-Exo, while 311 and 73 proteins were detected only in EC-Exo and SC-Exo, respectively. Bioinformatic analysis revealed that EC-Exo enriched proteins were involved in neurovascular function, while SC-Exo enriched proteins were related to lipid metabolism. Western blot analysis of 14 enriched proteins revealed that EC-Exo contained proteins involved in mediating endothelial function such as delta-like 4 (DLL4) and endothelial NOS (NOS3), whereas SC-Exo had proteins involved in mediating glial function such as apolipoprotein A-I (APOA1) and phospholipid transfer protein (PLTP). Collectively, the present study identifies differences in the cargo protein profiles of EC-Exo and SC-Exo, thus providing new molecular insights into their biological functions for the treatment of peripheral neuropathy.

## Introduction

Schwann cells are the most abundant cells in the peripheral nervous system (PNS) and play a vital role in the maintenance of peripheral nerve function [1, 2]. Microvascular endothelial cells maintain neurovascular function through crosstalk among endothelium, Schwann cells, and nerve fibers [3, 4]. Dysfunction of this communication is involved in the development of peripheral nerve damage [5, 6].

Exosomes are nano-size biovesicles (~30-200nm) released from nearly all cells and they play critical roles in mediating intercellular communication. Emerging evidence shows that exosomes have therapeutic effects on neurodegenerative diseases [7, 8]. We have demonstrated that exosomes derived from endothelial cells (EC-Exo) and Schwann cells (SC-Exo) ameliorate peripheral neuropathy caused by diabetes and chemotherapy [9, 10]. The therapeutic effect of exosomes on neurological diseases is likely impacted by transferring exosomal cargo biological materials into recipient cells, consequently leading to changes of recipient cell function [11, 12]. Exosomal cargo materials contain proteins, RNAs, and lipids, which are from the original cells. Although both EC-Exo and SC-Exo have therapeutic effects on peripheral neuropathy, the molecular mechanisms underlying the beneficial effects of these two exosomes may differ. Until now, only a few studies have investigated the protein profiles of EC-Exo and SC-Exo [13–16]. Knowledge of their cargo contents will provide new insights into the molecular mechanisms of these respective exosomes. This may permit further development of exosome-based treatment for neuropathy by engineering their respective molecular content, e.g. incorporating specific cargo proteins to enhance neurovascular and myelin function. In the present study, we thus analyzed protein profiles of EC-Exo derived from mouse dermal microvascular endothelial cells and of SC-Exo derived from Schwann cells.

## Materials and methods

### Primary cell culture

Primary mouse dermal microvascular endothelial cells were purchased from Cell Biologics (C57-6064), which were isolated from skin tissues of C57BL/6 mice. These cells are well characterized morphologically and phenotypically (VE-cadherin; AF1002; CD31/PECAM-1 positive). Primary Schwann cells were purchased from ScienCell (M1700-57), which were isolated from postnatal day 8 C57BL/6 mouse sciatic nerves. These cells exhibit Schwann cell phenotype marker proteins of S100, GFAP and CD9.

### Isolation of EC-Exo and SC-Exo

Mouse primary dermal microvascular endothelial cells and Schwann cells at passage 3–5 were cultured using complete culture medium (M168, cell biologics and 1701 ScienCell, respectively). When the cells reach to 60%~80% confluence, the exosome-depleted fetal bovine serum (FBS) medium (SF-4Z0-500, Cell Systems) was replaced and cultured for an additional 48 hours. The supernatant was then collected. Using a differentiation ultracentrifugation approach, exosomes were isolated from the supernatant according to our published protocol [10]. Briefly, the supernatant was passed through a 0.22 μm filter to remove dead cells and large debris. A 10,000g centrifugation for 30 min was performed to further remove small debris. Ultracentrifugation was performed at 100,000g (Optima XE-100 Ultracentrifuge, SW 32 Ti Rotor) for 2 hours and the pellet was resuspended with sterilized phosphate-buffered saline (PBS). The concentration and size distribution of exosomes were quantified using the NanoSight NS300 system (Malvern Panalytical). The exosomes were further verified by transmission electron microscopy (TEM, JEOL JEM- 1400). Western blot analysis with antibodies Alix, CD9, CD63, CD81 and Calnexin were used to confirm exosome marker proteins.

### Proteomics analysis

The mass spectrometry proteomics data have been deposited to the ProteomeXchange Consortium via the PRIDE [17] partner repository with the dataset identifier PXD041547 and 10.6019/PXD041547.

Two individual replicates of EC-Exo or SC-Exo were used for proteomics analysis and each EC-Exo and SC-Exo biological replicate was isolated from supernatant pooled from 3 independent cell cultures.

Total proteins in the exosomes were extracted according to published protocols [9, 10]. Briefly, the exosomes were lysed in 50 μl RIPA buffer with 1% protease inhibitor cocktail (Sigma-Aldrich) and incubated at 4°C for 30 min and followed by gentle mixing on ice for 15 min. The protein concentrations were determined by a bicinchoninic acid assay (BCA, Piece). The samples were submitted to the proteomics core facility of Wayne State University for exosomal cargo protein analysis. Briefly, 30 μg of each sample was heated at 95°C for 5 min with the addition of 2% lithium dodecyl sulfate. Samples were resuspended in 20 mM triethylammonium bicarbonate (TEAB) buffer, then reduced with 5 mM DL-Dithiothretol (DTT) and alkylated with 15 mM iodoacetamide (IAA) under standard conditions. Excess IAA was quenched with an additional 5 mM DTT. Next, the S-Trap precipitation protocol (Protifi) was performed, followed by an overnight digestion at 37°C in 40 mM TEAB and sequencing-grade trypsin (Promega). The next day, the peptides were separated by reversed-phase chromatography (Acclaim PepMap100 C18 column, Thermo Scientific), followed by ionization with the Nanospray Flex Ion Source (Thermo Scientific), and introduced into a Q Exactive mass spectrometer (Thermo Scientific). Abundant species were fragmented with high-energy collision-induced dissociation (HCID).

The raw data analysis was performed using Proteome Discoverer 2.4 (Thermo Scientific) which incorporated the Sequest algorithm (Thermo Scientific). The Uniprot_Mus_Compl_20181221 database was searched for mouse protein sequences and a reverse decoy protein database was run simultaneously for false discovery rate (FDR) determination. The data files were loaded into Scaffold (Proteome Software) for distribution.

Sequest was searched with a fragment ion mass tolerance of 0.02 Da and a parent ion tolerance of 10 PPM. Carbamidomethylation of cysteine was specified in Sequest as a fixed modification. Deamidation of asparagine and glutamine, oxidation of methionine, and acetylation of the n-terminus were specified in Sequest as variable modifications.

### Bioinformatics analysis

Protein stoichiometry in differences of EC-Exo and SC-Exo was analyzed using R programming (V4.2.1). Differential Enrichment analysis of Proteomics data (DEP) package (V1.16.00), general and standard approaches for analysis of proteomics, were applied for the differential protein analysis. Outliers from the protein profiles were eliminated from the analysis. First, the read count of each peptide was normalized using the variance stabilizing normalization (VSN) method. Then, the missing values from the protein profiles were identified and imputed with the k-nearest neighbor (KNN) approach. Proteins detected in both replicates of EC-Exo or SC-Exo were used for analysis. Next, a moderated t-tests built in the DEP package based on FDR calculated a statistical significance between EC-Exo and SC-Exo based on FDR determination. The log fold change >2 and p<0.05 were used to make a volcano visual plot. The differential proteins were separated into three subgroups: EC-Exo specific, SC-Exo specific, and differentially expressed based on their read count.

Protein enrichment analysis was performed on each subgroup using the Database for Annotation, Visualization and Integrated Discovery (DAVID) Bioinformatics Resources (V 6.8). Top gene ontology (GO) annotations including biological processes (BP), cellular components (CC), molecular function (MF) and Kyoto Encyclopedia of Genes and Genomes (KEGG) pathway for each subgroup were visualized by GraphPad Prime (V 9.0). For further the pathway analysis, Ingenuity Pathway Analysis (IPA, QIAGEN) database was used. The Canonical Pathways as well as Diseases and Biological Function tools from IPA Core analysis were performed on all significant proteins from each subgroup. The significant cut-off was set to Z-score > 1.3. The present study was not associated with cancer, so the cancer related pathways and biological functions were eliminated from the results. However, the pathways and functions that mediated cellular function and cell cycle were kept. All the significant proteins were identified and compared with ExoCarta database which is an online source specific on exosome protein profiles. The core proteins from each subgroup were identified based on their enrichment in the top GO terms and pathways and were validated by the Western Blot.

### Western blot analysis of selected proteins

EC-Exo and SC-Exo (n = 3/group) were homogenized in lysis buffer and the protein concentrations were determined using a bicinchoninic acid assay [10]. Western blot was performed according to published protocols [10]. Briefly, 30 μg of protein for each sample were subjected to electrophoresis and transferred to PVDF membrane. The membrane was incubated with primary antibodies followed by horseradish peroxidase (HRP)-conjugated secondary antibodies (1:1000) along with molecular weight markers. An enhanced chemiluminescence development kit was employed for detection. A list of individual primary and secondary antibodies used in the present study is provided in **S1 Table**. Statistical significance was determined using a student’s t test.

## Results

### Characterization of EC-Exo and SC-Exo

**Fig 1:**
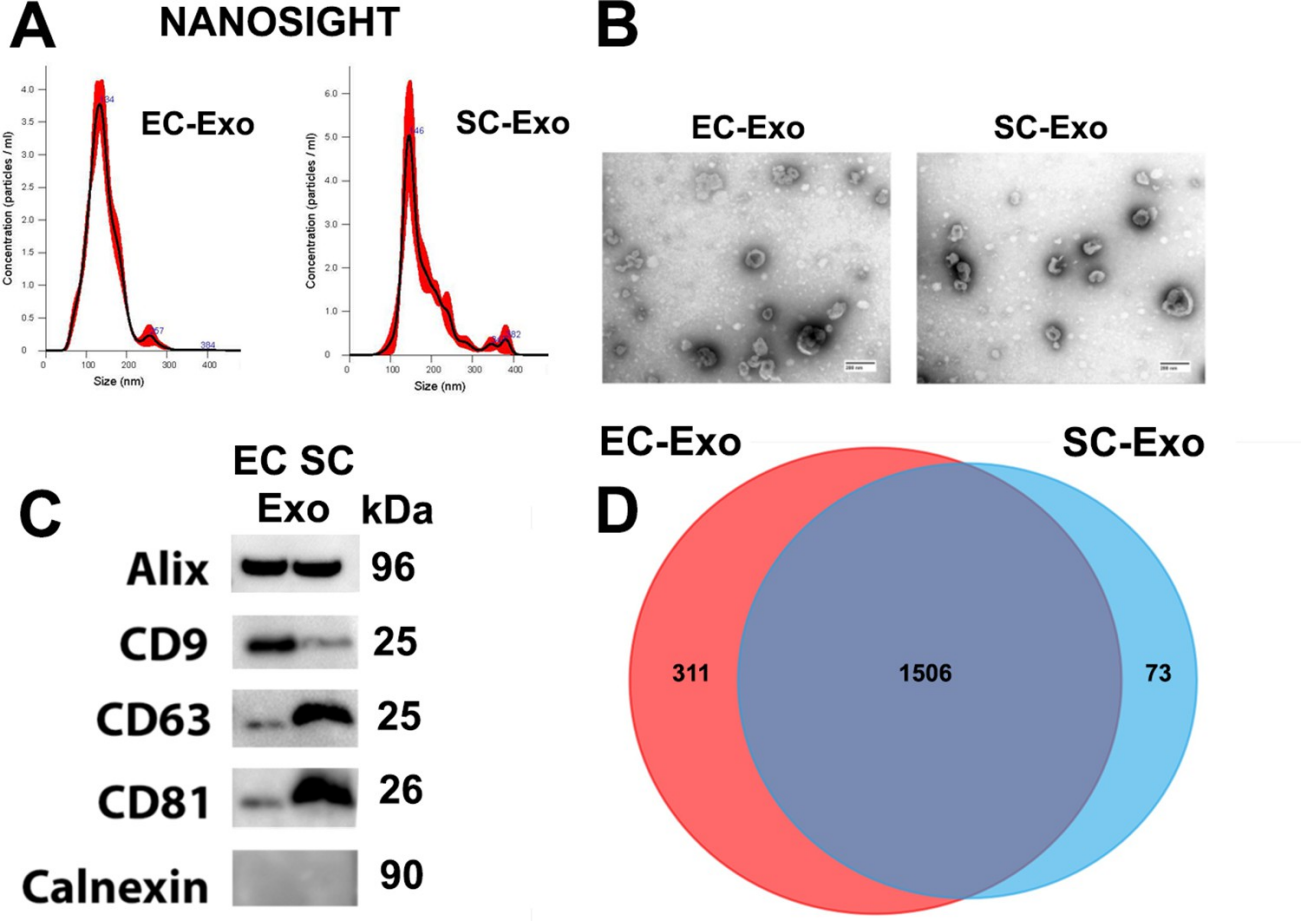
Characterization of EC-Exo and SC-Exo by (A) Particle size distribution analysis of EC-Exo and SC-Exo using the NanoSight analyzer, (B) Representative transmission electron micrographs (TEM) of EC-Exo and SC-Exo. Scale bar = 200nm. (C) Western blots analysis showing the expression of Alix, CD9, CD63, CD81 and Calnexin in EC-Exo and SC-Exo, respectively. (D) Venn diagram displaying common and unique proteins in EC-Exo and SC-Exo. EC-Exo, exosomes derived from endothelial cells. SC-Exo, exosomes derived from Schwann cells.

We first characterized EC-Exo and SC-Exo isolated from the supernatant of cultured ECs and SCs by a differential ultra-centrifugation approach [10]. Nanoparticle tracking analysis (NTA) revealed that the particle number and size of EC-Exo and SC-Exo were 3.0x10^9^/particles/ml and 3.6x10^9^/particles/ml with a mean size of 142±3.3 nm and 162±3.0 nm, respectively (**Fig 1A**). TEM analysis showed typical exosomal morphology (**Fig 1B**). Western blot analysis showed that EC-Exo and SC-Exo contained exosome marker proteins Alix, CD9, CD63 and CD81, but not Calnexin which is a negative control for an exosome protein marker (**Fig 1C**).

### Protein profiles in EC-Exo and SC-Exo

To examine stoichiometry differences in cargo protein compositions between EC-Exo and SC-Exo, we then performed a proteome analysis by means of label-free quantitative LC–MS.

Protein identifications were accepted if they had greater than 99.0% probability to achieve a false discovery rate (FDR) less than 1.0% and contained at least 2 identified peptides. Based on these criteria, there were 1,817 and 1,579 proteins in EC-Exo and SC-Exo, respectively. Among the detected proteins, approximately 97% proteins (1,766 in EC-Exo and 1,544 in SC-Exo) have been reported in the exosome protein database, Exocarta. Although there were 1,506 proteins that were shared between EC-Exo and SC-Exo, there were 311 and 73 proteins that were highly enriched in EC-Exo and in SC-Exo, respectively (**Fig 1D**), which reflects the different nature of their parent cells between endothelial cells and Schwann cells.

### Bioinformatics analysis of EC-Exo enriched proteins

**Fig 2:**
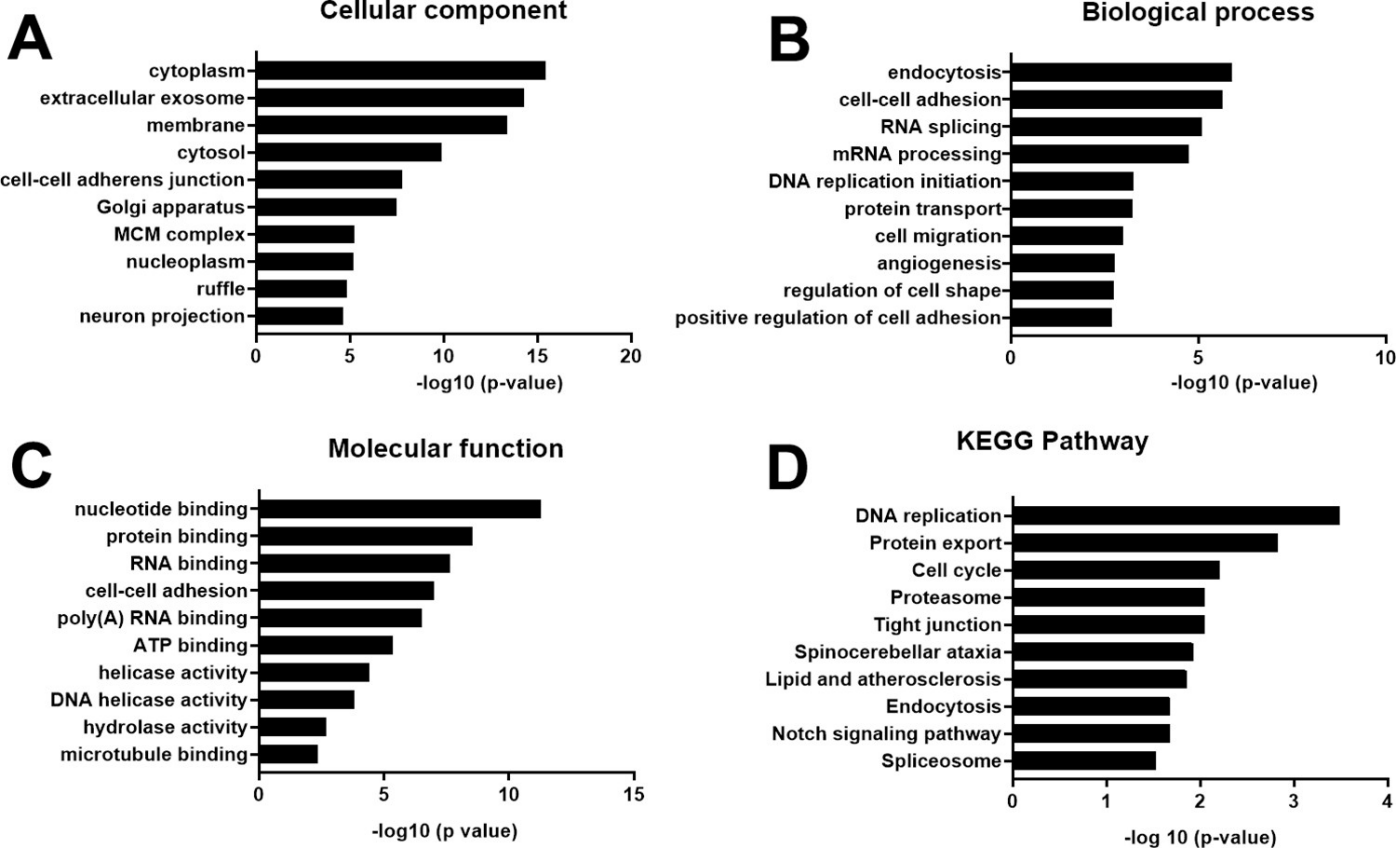
Analysis of EC-Exo enriched proteins. GO functional analysis showed the 10 most significantly (p<0.05) enriched proteins in cellular component (**A**), biological process (**B**), molecular function **(C).** KEGG pathway enrichment analysis showed pathways that these proteins are involved (**D**). The x axis shows the negative of the log-base-10 of the p- value.

To further characterize EC-Exo and SC-Exo enriched proteins, we first performed GO analysis of EC-Exo enriched proteins (**S2 Table**). The top 10 GO terms ranked according to their significance level (p<0.05) are listed in **Fig 2**. Functional annotation to GO parent terms “cellular components” assigned the largest fraction of identified proteins to the category of “extracellular” exosome (**Fig 2**), indicating that our procedures were reliable and effective to isolate exosomes. Additionally, cellular component identified proteins that were significantly enriched in cytoplasm (**Fig 2A**). Cytoplasmic proteins are mainly involved energy production, metabolism, and protein biosynthesis [18]. The biological processes are mainly related to endocytosis and cell-cell adhesion, as well as angiogenesis (**Fig 2B**). In terms of molecular functions, proteins were enriched in nucleotide binding and protein binding (**Fig 2C**). Binding proteins on the exosome surface may facilitate exosomes to target recipient cells [19].

In addition, the KEGG pathway enrichment analysis revealed that these proteins are related to Notch signaling and tight junction pathways (**Fig 2D**). The Notch signaling pathway mediates angiogenesis and axonal regeneration [20, 21], while the tight junction pathway plays a crucial role in angiogenesis and control the permeability of blood vessels [22].

**Fig 3:**
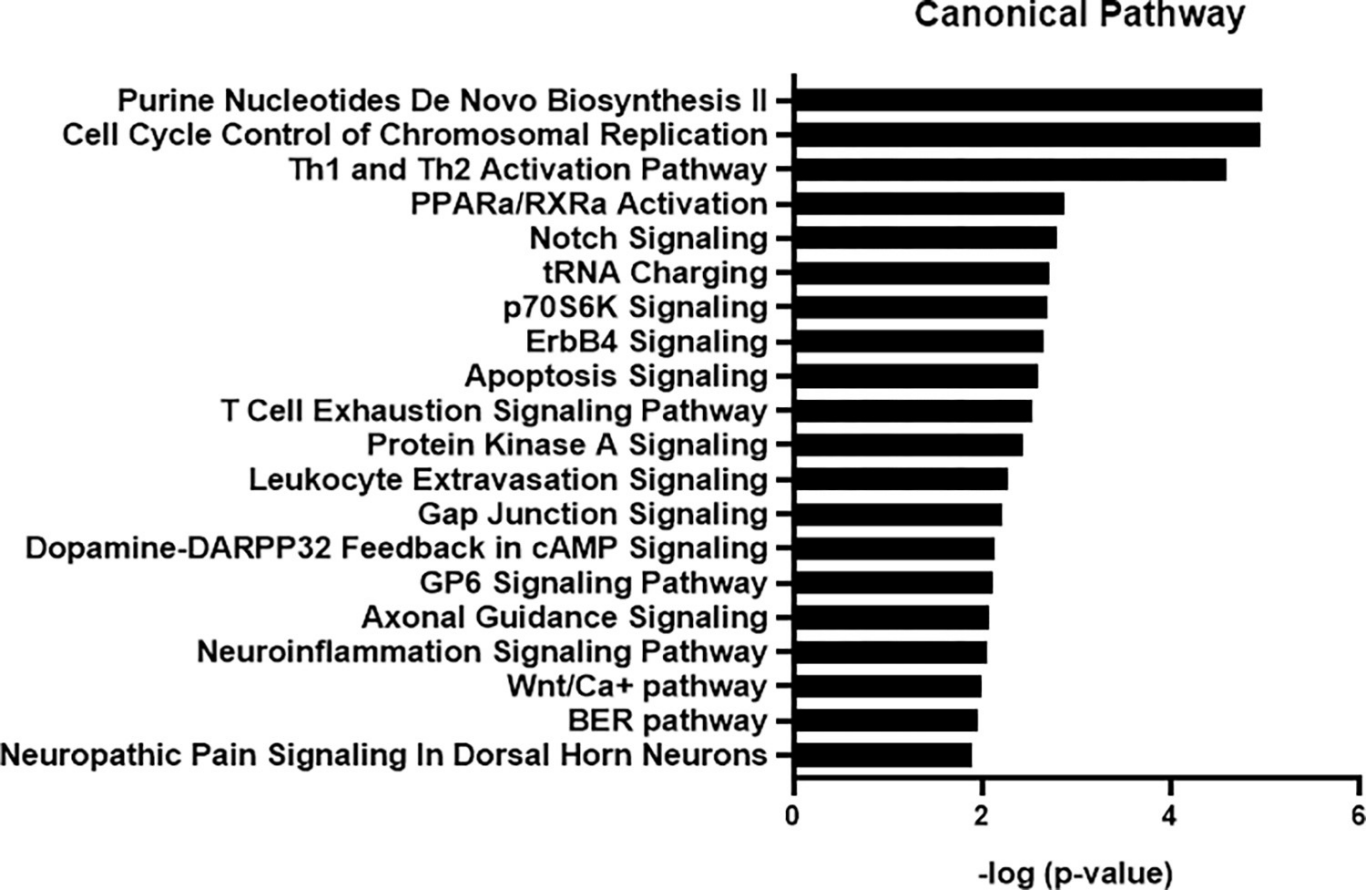
Canonical pathway analysis of EC-Exo enriched proteins using IPA. The top 20 most significant pathways are presented. The x-axis shows the negative log of p-value. IPA, ingenuity pathway analysis.

Using IPA, we further analyzed key signaling pathways associated with EC-Exo enriched proteins. A total of 81 enriched canonical pathways were identified in these proteins by using the -log(p-value) >1.3 threshold. The top 20 signaling pathways are shown in **Fig 3**. The signaling pathways involved in angiogenesis and vascular function such as T helper cell type 1 (Th1)/T helper cell type II (Th2) and Peroxisome Proliferator Activated Receptor Alpha (PPARa)/Retinoid X Receptor Alpha (RXRa) activation and Notch signaling were highly ranked. Th1/Th2 cytokines control angiogenesis, while PPARa/RXRa signaling regulates vascular and inflammatory responses [23]. In addition, the protein kinase A (PKA) signaling pathway is involved in regulation of synaptic plasticity and vascular tone.

**Table 1** lists EC-Exo enriched proteins that are involved in vascular function such as angiogenesis and vasculogenesis. These proteins included proteins in the Notch pathway, delta-like protein 4 (DLL4) and presenilin-1 (PSEN1), as well as stromal cell-derived factor 1 (CXCL12), Ephrin-B2 (EFNB2), matrix metalloproteinase-14 (MMP14), protein kinase C alpha type (PRKCA) and semaphorin-3C (SEMA3c). In addition to vascular function, these EC-Exo enriched proteins are also involved in axonal guidance signaling and growth (**Table 2**).

**Table 1:** Exosome proteins associated with vascular function.

**EC-Exo enriched proteins**		
**Protein ID**	**Gene name**	**Protein name**
P15116	CDH2	Cadherin-2
Q91WQ3	YARS1	Tyrosine—tRNA ligase, cytoplasmic
Q8VCC6	CCM2	Cerebral cavernous malformations protein 2
P48678	LMNA	Prelamin-A
Q9JI71	DLL4	Delta-like protein 4
Q60805	MERTK	Tyrosine-protein kinase Mer
Q62181	SEMA3C	Semaphorin-3C
P52795	EFNB2	Ephrin-B2
Q811D0	DLG1	Disks large homolog 1
Q9Z0J1	RECK	Reversion-inducing cysteine-rich protein with Kazal motifs
P83741	WNK	Serine/threonine-protein kinase WNK1
Q60751	IGF1R	Insulin-like growth factor 1 receptor
P28862	MMP3	Stromelysin-1
Q08857	CD36	Platelet glycoprotein 4
Q01102	SELP	P-selectin
Q9JKK1	STX6	Syntaxin-6
Q8K019	BCLAF1	Bcl-2-associated transcription factor 1
P16382	IL4R	Interleukin-4 receptor subunit alpha
Q9D154	SERPINE1	Plasminogen activator inhibitor 1
P70372	ELAV1	ELAV-like protein 1
Q61288	ACVR1	Activin receptor type-1
P23242	GJA1	Gap junction alpha-1 protein
Q3MI99	CCBE1	Collagen and calcium-binding EGF domain-containing protein 1
P40224	CXCL12	Stromal cell-derived factor 1
O08599	STXBP2	Syntaxin-binding protein 2
P98156	VLDLR	Very low-density lipoprotein receptor
Q8CFG0	SULF2	Extracellular sulfatase Sulf-2
Q62433	NDRG1	Protein NDRG1
Q8BVF7	APH1A	Gamma-secretase subunit APH-1A
Q8R4G6	MGAT5	Alpha-1,6-mannosylglycoprotein 6-beta-N-acetylglucosaminyltransferase A
P49769	PSEN1	Presenilin-1
Q9R0C8	VAV3	Guanine nucleotide exchange factor VAV3
Q9Z1B3	PLCB1	1-phosphatidylinositol 4,5-bisphosphate phosphodiesterase beta-1
Q8R3B1	PLCD1	1-phosphatidylinositol 4,5-bisphosphate phosphodiesterase delta-1
Q9QUH0	GLRX	Glutaredoxin-1
Q62443	NPTX1	Neuronal pentraxin-1
P31230	AIMP1	Aminoacyl tRNA synthase complex-interacting multifunctional protein 1
Q64455	PTPRJ	Receptor-type tyrosine-protein phosphatase eta
Q8VE98	CD276	CD276 antigen
P11103	PARP1	Poly [ADP-ribose] polymerase 1
P58022	LOXL2	Lysyl oxidase homolog 2
P20444	PRKCA	Protein kinase C alpha type
P56546	CTBP2	C-terminal-binding protein 2
P27808	MGAT1	Alpha-1,3-mannosyl-glycoprotein 2-beta-N-acetylglucosaminyltransferase
P41241	CSK	Tyrosine-protein kinase CSK
P16110	LGALS3	Galectin-1
O89110	CASP8	Caspase-8
P53690	MMP14	Matrix metalloproteinase-14
P09470	ACE	Angiotensin-converting enzyme
P70313	NOS3	Nitric oxide synthase, endothelial
Q9CQI3	GMFB	Glia maturation factor beta
Q9WUD1	STUB1	STIP1 homology and U box-containing protein 1
**SC-Exo enriched proteins**		
**Protein ID**	**Gene name**	**Protein name**
P23927	CRYAB	Alpha-crystallin B chain
Q8R0X7	SGPL1	Sphingosine-1-phosphate lyase 1
Q99K41	EMILIN1	EMILIN-1
P47877	IGFBP2	Insulin-like growth factor-binding protein2
Q8C4U3	SFRP1	Secreted frizzled-related protein 1
Q9R045	ANGPTL2	Angiopoietin-related protein 2
P35441	THBS1	Thrombospondin-1
Q9R182	ANGPTL3	Angiopoietin-related protein 3
O55188	DMP1	Dentin matrix acidic phosphoprotein 1
O35474	EDIL3	EGF-like repeat and discoidin I-like domain-containing protein 3
Q61738	ITGA7	Integrin alpha-7
Q04207	RELA	Transcription factor p65
P14602	HSPB1	Heat shock protein beta-1
Q9WVJ9	EFEMP2	EGF-containing fibulin-like extracellular matrix protein 1
O35945	ALDH1A7	Aldehyde dehydrogenase, cytosolic 1
P37889	FBLN2	Fibulin-2
P18242	CATHEPSIN	Cathepsin

Exosome proteins associated with vascular function. The table shows a list of proteins that are enriched in EC-Exo and SC-Exo.

**Table 2:** Exosome proteins associated with neural function.

**EC-Exo enriched proteins**		
**Protein ID**	**Gene name**	**Protein name**
Q7TT50	CDC42B	Serine/threonine-protein kinase MRCK beta
P52800	EFNB2	Ephrin-B2
F8VQB6	MYO10	Unconventional myosin
P40224	CXCL12	Stromal cell-derived factor 1
P15116	CDH2	Cadherin-2
P98156	VLDLR	Very low-density lipoprotein receptor
Q62443	NPTX1	Neuronal pentraxin-1
P35285	RAB22A	Ras-related protein Rab-22A
Q62448	EIF4G2	Eukaryotic translation initiation factor 4 gamma 2
P28740	KIF2A	Kinesin-like protein KIF2A
Q60751	IGF1R	Insulin-like growth factor 1 receptor
P11103	PARP1	Poly [ADP-ribose] polymerase 1
Q9WV80	SNX1	Sorting nexin-12
Q9ES28	ARHGEF7	Rho guanine nucleotide exchange factor 7
Q8BL66	EEA1	Early endosome antigen 1
P22777	SERPINE1	Plasminogen activator inhibitor 1
O70493	SNX12	Sorting nexin-12
B0V2N1	PTPRS	Receptor-type tyrosine-protein phosphatase S
P49769	PSEN1	Presenilin-1
P23242	GJA1	Gap junction alpha-1 protein
Q60780	GAS7	Growth arrest-specific protein 7
Q9ES28	ARHGEF7	Rho guanine nucleotide exchange factor 7
Q03137	EPHA4	Ephrin type-A receptor 4
O88447	KLC1	Kinesin light chain 1
P53690	MMP14	Matrix metalloproteinase-14
P28862	MMP3	Matrix metalloproteinase-3
Q8VD65	PIK3R4	Phosphoinositide 3-kinase regulatory subunit 4
Q9Z1B3	PLCB1	1-phosphatidylinositol 4,5-bisphosphate phosphodiesterase beta-1
Q8R3B1	PLCD1	1-phosphatidylinositol 4,5-bisphosphate phosphodiesterase delta-1
Q8CIH5	PLCG2	1-phosphatidylinositol 4,5-bisphosphate phosphodiesterase gamma-2
P20444	PRKCA	Protein kinase C alpha type
Q62181	SEMA3C	Semaphorin-3C
Q76KF0	SEMA6D	Semaphorin-6D
Q6PHZ2	CAMK2D	Calcium/calmodulin-dependent protein kinase type II subunit delta
Q80X90	FLNB	Filamin-B
**SC-Exo enriched proteins**		
**Protein ID**	**Gene name**	**Protein name**
O35945	ALDH1a7	Aldehyde dehydrogenase, cytosolic 1
O08917	FLOT1	Flotillin-1
P18872	GNAO1	Guanine nucleotide-binding protein G(o) subunit alpha
Q62059	VCAN	Versican core protein
O08989	MRAS	Ras-related protein M-Ras
Q07235	SERPINE2	Plasminogen activator inhibitor 1
P35441	THBS1	Thrombospondin-1
Q04207	RELA	Prolow-density lipoprotein receptor-related protein 1
P18872	GNAO1	Guanine nucleotide-binding protein G(o) subunit alpha

Exosome proteins associated with neural function. The table shows a list of proteins that are enriched in EC-Exo and SC-Exo.

### Bioinformatics analysis of SC-Exo enriched proteins

**Fig 4:**
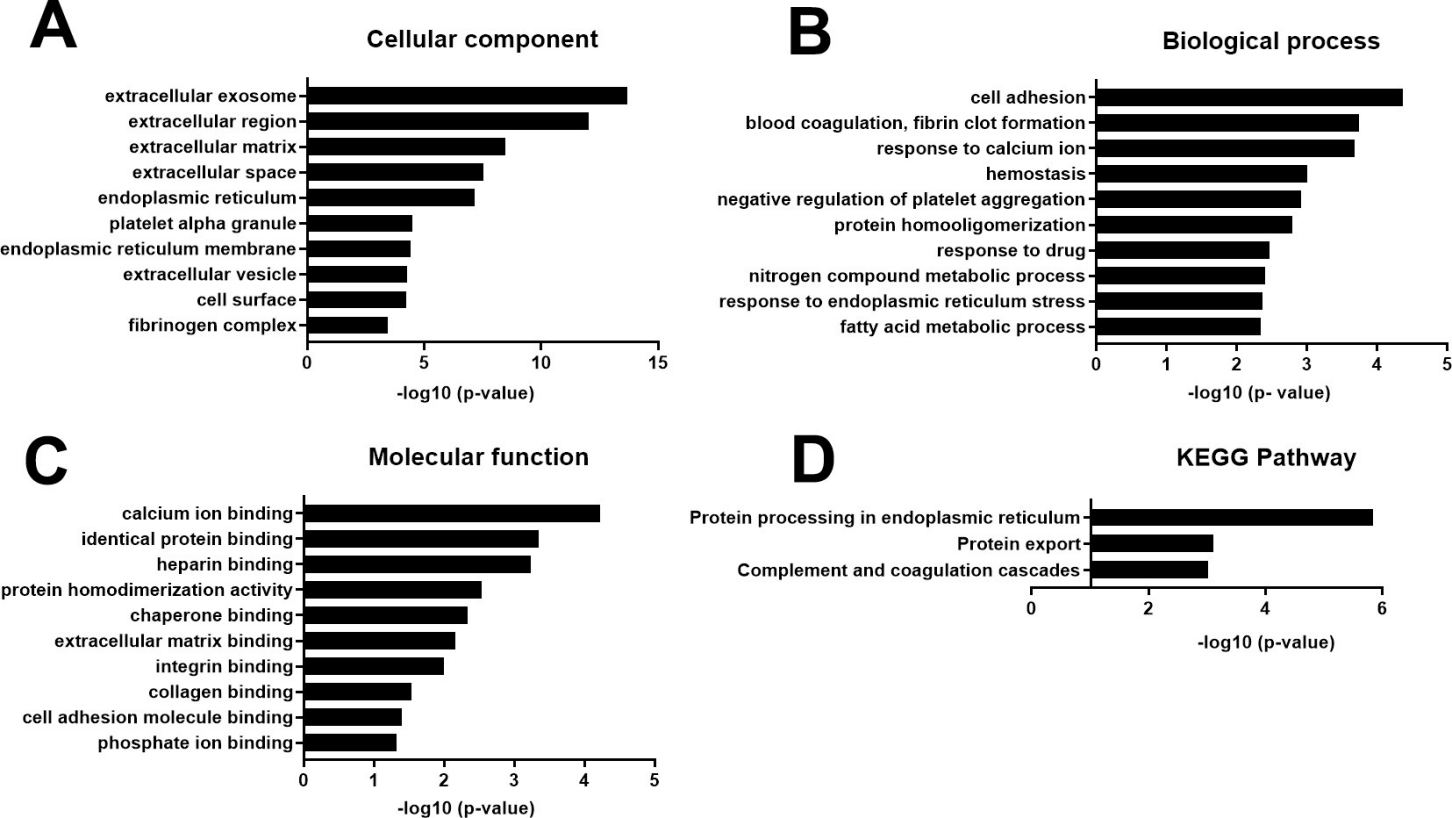
Analysis of EC-Exo enriched proteins. GO enrichment analysis showed the 10 most significantly (p<0.05) enriched proteins in cellular component (**A**), biological process (**B**), molecular function (**C**). KEGG pathway enrichment analysis showed pathways that these proteins are involved (**D**). The x-axis shows the negative of the log-base-10 of the p- value.

Compared to EC-Exo, there were 73 enriched proteins in SC-Exo (**S3 Table**). GO analysis of cellular components showed that enriched SC-Exo proteins were related to extracellular exosome, extracellular region, and extracellular space (**Fig 4A)**. These proteins significantly enriched biological processes and molecular function were cell adhesion, response to calcium ion, and calcium ion binding proteins (**Fig 4B and 4C**), which is in line with the supportive role of SCs in peripheral nerve function [24, 25]. The KEGG pathway analysis showed that these proteins are involved in the endoplasmic reticulum pathways via protein processing. The endoplasmic reticulum pathway is critical for Schwann cells to synthesize myelin protein and to maintain myelin structure (**Fig 4D**) [26].

**Fig 5:**
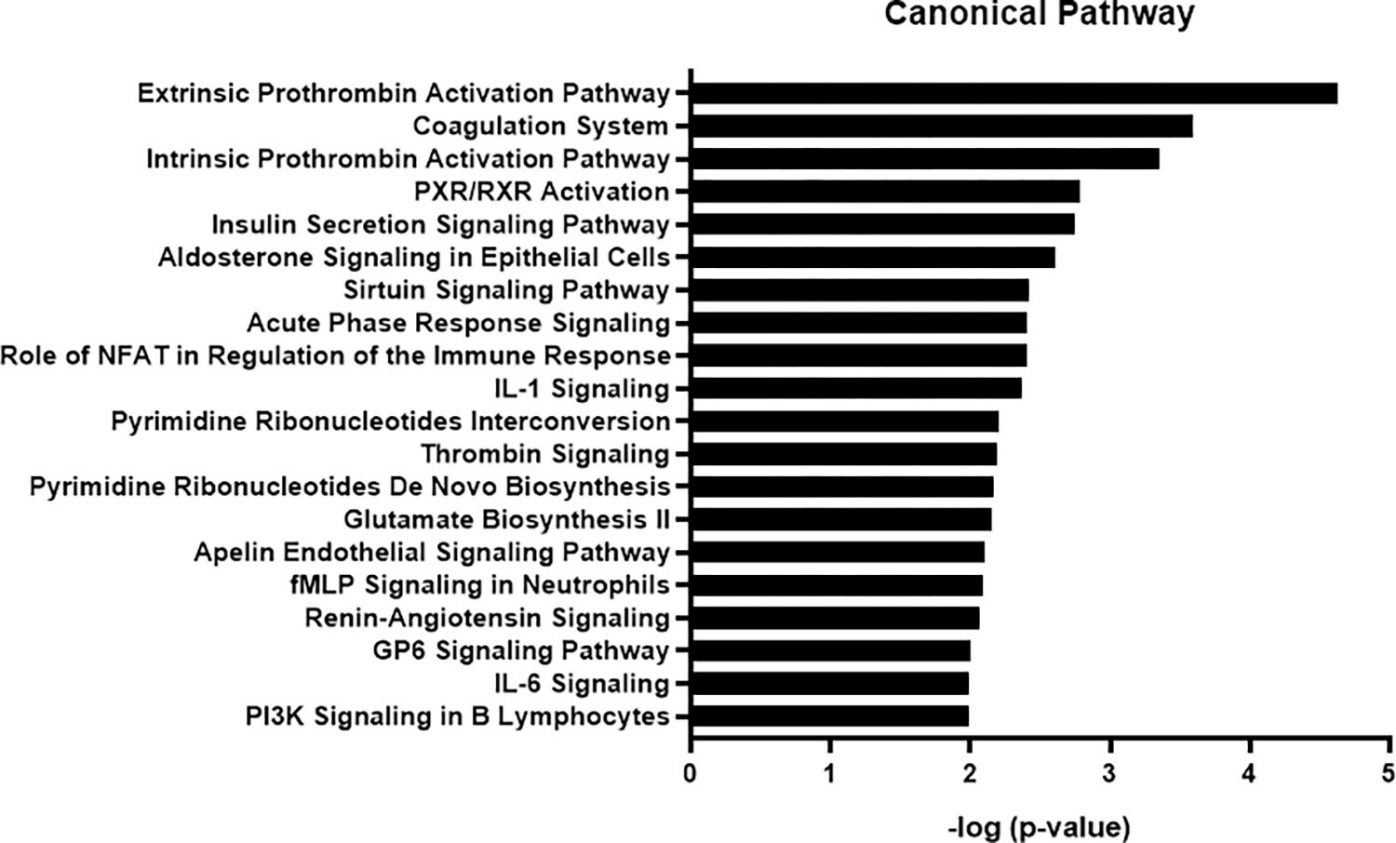
Canonical pathway analysis of SC-Exo enriched proteins using IPA. The top 20 most significant pathways are presented. The x-axis shows the negative log of p- value.

IPA showed that the top 20 canonical pathways in SC-Exo enriched proteins include the insulin secretion pathway and pregnane X receptor (PXR)/retinoid x receptor (RXR) signaling (**Fig 5**). Disrupting insulin signaling such as insulin-like growth factor-binding protein 2 (IGFBP2) in SCs, impairs myelination and induces a sensory neuropathy [27]. RXRr accelerates remyelination [28]. In addition, the PI3K signaling pathway modulates axonal outgrowth and myelination [29]. Moreover, the extrinsic/intrinsic prothrombin activation pathways regulate Schwann cell supported neuronal regeneration [30]. Furthermore, interleukin-1 regulates proliferation and differentiation of oligodendrocyte progenitor cells [31].

In contrast to EC-Exo, SC-Exo were enriched with proteins involved in metabolism, synthesis, and transportation of lipids. These proteins included glia-derived nexin (SERPINE2), cytochrome b5 (CYB5A), angiopoietin-related protein 3 (ANGPTL3), adipocyte enhancer-binding protein 1(AEBP1) and IGFBP2 (**Table 3**). Additionally, SC-Exo were also enriched with proteins involved in peripheral nerve fiber growth and axonal guidance, such as flotillin-1 (FLOT1), thrombospondin-1 (THBS1), Ras-related protein M-Ras (MRAS) and guanine nucleotide-binding protein G(o) subunit alpha (GNAO1) (**Table 2**). Thus, these data suggest that SC-Exo could mediate myelination and axonal growth.

**Table 3:** Exosome proteins associated with lipid metabolism process.

**EC-Exo enriched proteins**		
**Protein ID**	**Gene name**	**Protein name**
P41233	ABCA1	ATP-binding cassette sub-family A member 1
Q64343	ABCG1	ATP-binding cassette sub-family G member 1
P47856	GFPT1	Glutamine—fructose-6-phosphate aminotransferase [isomerizing] 1
P48678	LMNA	Prelamin-A/C
P06795	ABCB1	Multidrug resistance protein 1B
Q9Z1B3	PLCB1	1-phosphatidylinositol 4,5-bisphosphate phosphodiesterase beta-1
Q8R3B1	PLCD1	1-phosphatidylinositol 4,5-bisphosphate phosphodiesterase delta-1
Q62077	PLCG2	1-phosphatidylinositol 4,5-bisphosphate phosphodiesterase gamma-1
Q08857	CD36	Platelet glycoprotein 4
P11103	PARP1	Poly [ADP-ribose] polymerase 1
P98156	VLDLR	Very low-density lipoprotein receptor
P70372	ELAVL1	ELAV-like protein 1
Q9JIZ9	PLSCR3	Phospholipid scramblase 3
P62331	ARF6	ADP-ribosylation factor 6
**SC-Exo enriched proteins**		
**Protein ID**	**Gene name**	**Protein name**
**Q9Z0K8**	VNN1	Pantetheinase
Q640N1	AEBP1	Adipocyte enhancer-binding protein 1
Q8R0X7	SGPL1	Sphingosine-1-phosphate lyase 1
Q8BLF1	NCEH1	Neutral cholesterol ester hydrolase 1
P97742	CPT1A	Carnitine O-palmitoyltransferase 1, liver isoform
Q9Z0J0	NPC2	NPC intracellular cholesterol transporter 2
Q9R045	ANGPTL1	Angiopoietin-related protein 2
Q04207	RELA	Transcription factor p65
Q9QXP7	C1QTNF1	Complement C1q tumor necrosis factor-related protein 1
P56395	CYB5A	Cytochrome b5
Q8R0X7	SGPL1	Sphingosine-1-phosphate lyase 1
O35945	ALDH1A7	Aldehyde dehydrogenase, cytosolic 1
P51660	HSD17B4	Peroxisomal multifunctional enzyme type 2
Q80TA6	MTMR12	Myotubularin-related protein 12
Q07235	SERPINE2	Glia-derived nexin
P47877	IGFBP2	Insulin-like growth factor-binding protein 2
Q9Z0J0	NPC2	NPC intracellular cholesterol transporter 2
Q9R182	ANGPTL3	Angiopoietin-related protein 3
Q99L43	CDS2	Phosphatidate cytidylyltransferase 2
O08917	FLOT1	Flotillin-1
Q640N1	AEBP1	Adipocyte enhancer-binding protein 1
Q99KQ4	NAMPT	Nicotinamide phosphoribosyltransferase
Q99LJ1	FUCA1	Tissue alpha-L-fucosidase
O08710	TG	Thyroglobulin
O35474	EDIL3	EGF-like repeat and discoidin I-like domain-containing protein 3
Q64521	GPD2	Glycerol-3-phosphate dehydrogenase, mitochondrial
Q00623	APOA1	Apolipoprotein A-I
P50428	ARSA	Arylsulfatase A
Q60931	VDAC3	Voltage-dependent anion-selective channel protein 3
P26443	GLUD1	Glutamate dehydrogenase 1
P18242	CATHEPSIN	Cathepsin
P11276	FN1	Fibronectin
P55065	PLTP	Phospholipid transfer protein

Exosome proteins associated with lipid metabolism. The table shows a list of proteins that are enriched in EC-Exo and SC-Exo.

### Bioinformatics analysis of the common proteins between EC-Exo and SC-Exo

**Fig 6:**
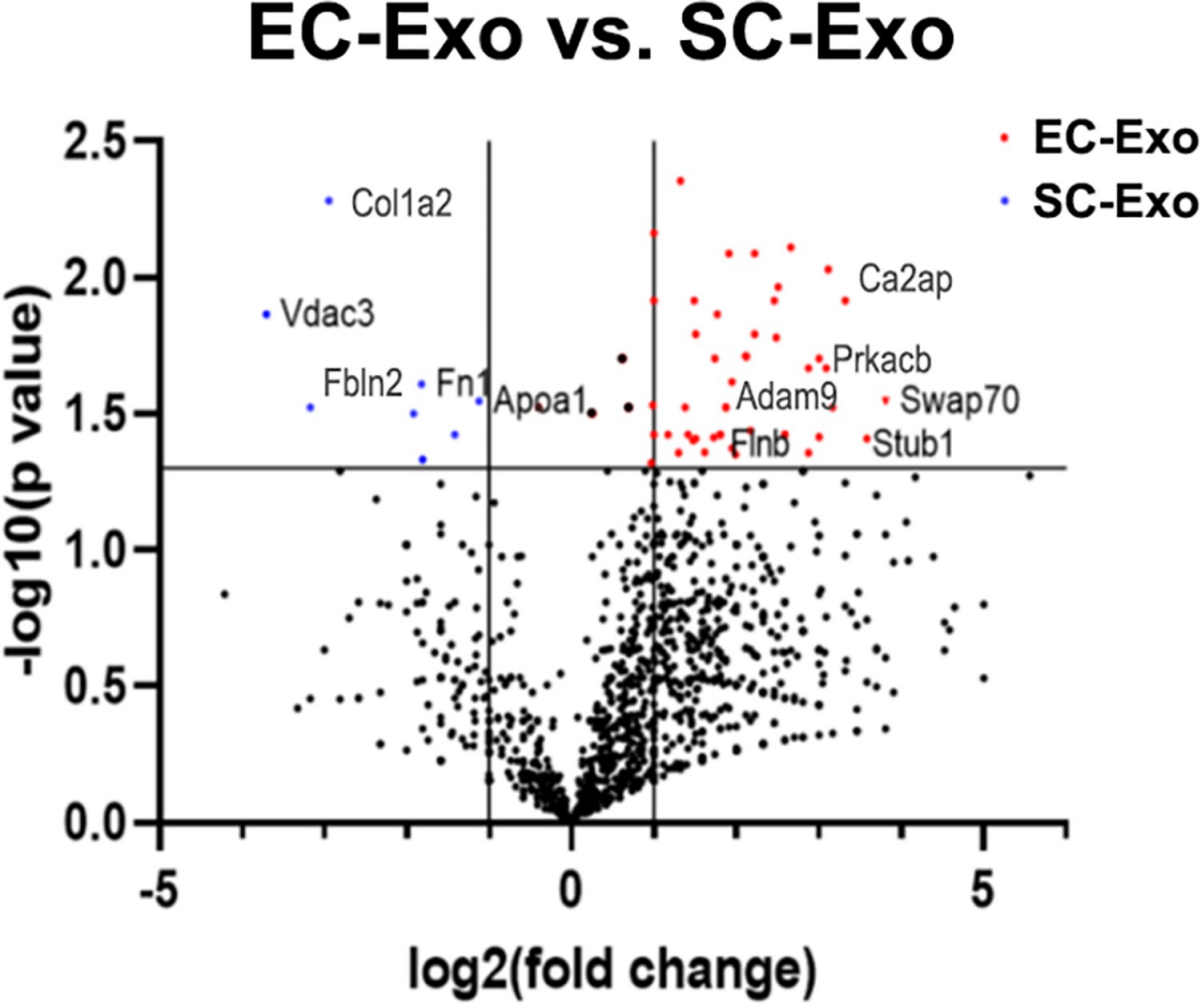
Volcano plot showing p-values (-log 10) versus protein ratio of (log2 EC-Exo vs.SC-Exo). Red: EC-Exo abundant proteins (fold-change>2, p-value<0.05), Blue: SC-Exo abundant proteins (fold-change <-2, p-value <0.05), grey: no significant change. A few selected differentially abundant proteins are labeled.

We then examined protein stoichiometry for differences among shared 1,506 common proteins between EC-Exo and SC-Exo. We found 47 and 8 abundant proteins in EC-Exo and in SC-Exo than in SC-Exo and EC-Exo, respectively, based on a threshold of p<0.05 with two-fold change (**Fig 6**, **S4 and S5 Tables**). The EC-Exo abundant proteins included disintegrin and metalloproteinase domain-containing protein 9 (ADAM9), CD2-associated protein (CD2AP), and cAMP-dependent protein kinase catalytic subunit beta (PRKACB), which mediate axon guidance and neurovascular function. There were multiple glycoproteins in SC-Exo that regulate myelin sheath formation, maintenance and degeneration, such as apolipoprotein A-I (APOA1), collagen alpha-2(I) chain (COL1A2), fibulin-2 (FBLN2), fibronectin (FN1), and cation-independent mannose-6-phosphate receptor (IGF2R).

### Western blot analysis to confirm the result of the proteomic experiment

**Fig 7:**
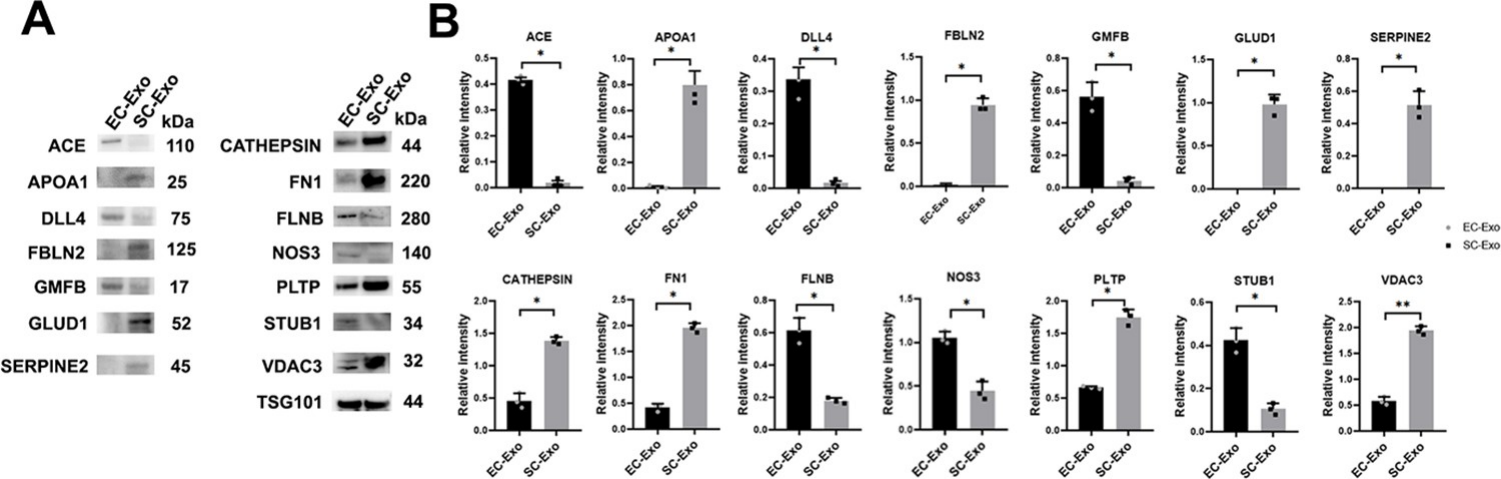
Western blot analysis of selective enriched proteins in EC-Exo and SC-Exo. Representative Western blot images (**A**) and their relatively quantitative data (**B**) showed protein levels in EC-Exo and SC-Exo. TSG101 protein was used as a reference protein. n = 3/group. Data represent mean±SE. *p-value <0.01.

Using Western blot analysis, we further measured 20 proteins selected based on differences between EC-Exo and SC-Exo. Among them, Western blot (**Fig 7, S1 Raw images)** confirmed EC-Exo relatively enriched proteins: angiotensin-converting enzyme (ACE), delta-like protein 4 (DLL4), glia maturation factor-β (GMFB), filamin-B (FLNB), endothelial nitric oxides synthase (NOS3), and STIP1 homology and U box-containing protein 1 (STUB1). For SC-Exo relatively enriched proteins were APOA1, FBLN2, SERPINE2, glutamate dehydrogenase 1 (GLUD1), CATHEPSIN, FN1, phospholipid transfer protein (PLTP). and Voltage-dependent anion-selective channel protein 3 (VDAC3).

## Discussion

The present study demonstrated that EC-Exo and SC-Exo have different protein cargo profiles, with EC-Exo enriched proteins involved in vascular function and SC-Exo enriched proteins involved in regulation of myelin related lipid metabolism, while cargo proteins shared by EC-Exo and SC-Exo could modulate axonal guidance and growth. These data provide evidence to support that EC-Exo and SC-Exo contribute to the roles of endothelial and Schwann cells in the growth and maintenance of peripheral nerves.

Comprehensive and comparative analysis of the composition and function of exosomal cargo proteins derived from healthy mouse endothelial cells and Schwann cells have not been reported. Mouse dermal microvascular endothelial cells are major components of skin blood vessels and provide nutrients to the epidermal nerve tissue, and closely mimic endothelial cell function in the PNS. A detailed comparative analysis between the two types of exosomes may provide increased insight into their biological function, and is critical for treatment of peripheral nerve damage, such as peripheral neuropathy. Dysfunction of communication between endothelial cells and Schwann cells is involved in the development of peripheral nerve damage [5, 6]. We and others have demonstrated that individual EC-Exo and SC-Exo treatment have therapeutic effects on peripheral neuropathy [9, 32]. The present study also provides information to potentially generate engineered exosomes by combining specific cargo proteins which regulate neurovascular and myelin functions from EC-Exo and SC-Exo. This may further advance exosome-based treatment for neuropathy.

The present study is consistent with and extends previous proteomics findings of exosomes derived from endothelial and Schwann cells [13, 15]. Wei et.al reported that exosomes derived from rat primary Schwann cells contain 12 proteins that mediate neuronal function, in which 11 of the 12 proteins were detected by the present study [13]. Li et.al revealed abundant levels of TGFβ2 and Octamer-binding transcription factor 4 in EVs isolated from the rat spontaneously immortalized Schwann cell line RSC96 [33]. Boyer et.al found that EVs derived from rat aortic endothelial cells contain proteins that modulate smooth muscle cell phenotype and protein synthesis [15].

Within EC-Exo enriched proteins, we observed that several key signaling pathways are related to angiogenesis, vasculogenesis, and axonal growth, which may contribute to the functional role of ECs. For example, several proteins were found to associate with the Notch signaling pathway. DLL4 is a transmembrane ligand for Notch receptors and endothelial DLL4 deficiency impairs arterial relaxation [34]. DLL4-containing EC exosomes participate in angiogenesis through interaction with recipient endothelial cells [35]. Of interest, NOS3 is a regulator of Notch signaling, and plays an important role in regulating vascular relaxation and blood flow by activating the soluble guanylate cyclase (sGC)-cGMP-PKG pathway and impairing vascular redox environment [36, 37].

In addition, several EC-Exo abundant proteins, such as ADAM9, CD2AP and PRKACB are related to axon guidance and growth. Filamin-B (FLNB) are expressed in endothelial cells and play an essential role in vascular development and angiogenesis [38]. Moreover, Glia maturation factor beta (GMFB) is a growth factor for both glia and neurons. It stimulates axon regeneration in transected rat sciatic nerve [39]. We speculate that the expression of these enriched proteins in EC-Exo may improve the microenvironment and enhance neurovascular remodeling in peripheral nerve damage.

SC-Exo have been mainly evaluated in the context of peripheral nerve repair [40–42]. We found that the enriched proteins in SC-Exo were mainly annotated to the extracellular exosome, calcium ion binding, and cell adhesion which strongly influence nerve regeneration and myelination [42, 43]. The enriched signaling pathway is involved in myelination and axonal regeneration, such as the insulin secretion pathway and the PI3K signaling pathway. Insulin signaling is essential for SC myelination by activating the PI3K/AKT pathway and lipid metabolism [27]. GLUD1 is a mitochondrial matrix enzyme and involved in insulin secretion [44].

SC-Exo enriched proteins were associated with lipid metabolism, including synthesis and transport of lipid. Myelin contains a high proportion of lipids [45]. The lipid synthesis and fatty acid are required for myelination and peripheral nerve function [27, 45]. Peripheral nerve myelin is affected in lipid metabolism disorder [46]. APOA1 is expressed in myelinating sciatic nerve and involved in myelin synthesis by the local transport of lipids [47]. It also increases neurite outgrowth and neuronal regeneration by restricting inflammatory response and enhances angiogenesis by synthesizing cell surface ATP [48]. Moreover, PLTP, a crucial modulator of lipoprotein (HDL) metabolism, may be involved in the maintenance of the functional and structural integrity of myelin and regulates axonal guidance and sprouting [49, 50]. SC-Exo enriched proteins are involved in nerve regeneration, such as cathepsins and serpine2. Others have shown that SC-Exo increase axonal growth of DRG neurons and promote regeneration of damaged peripheral nerve [32, 40, 51]. Fibronectin, a major extracellular matrix, regulates remyelination and promotes Schwann cell growth. Loss of fibronectin in SCs impairs their directional migration affecting the alignment of the axons [52]. Furthermore, VDAC3 may increase the efficiency of bioenergetic metabolism and protect mitochondria from oxidative stress which are critical for proper peripheral nerve function [53]. We thus speculate that lipid- and axon-related proteins in the SC-Exo cargo may enhance myelin, and axonal formation and function.

The present study revealed that EC-Exo and SC-Exo shared many proteins related to axonal guidance and nerve growth, suggesting that these cargo proteins could contribute to the therapeutic effect of EC-Exo and SC-Exo on peripheral neuropathy [9, 32].

The present study has limitations including a sample size with two replicates of each proteomics analysis and functional analysis, although each individual exosome sample was isolated from the supernatant pooled from three biological replicates. Our data also suggest that protein stoichiometry between different exosome cargo based on proteomics analysis needs to be further confirmed using Western blot analysis or other more quantitative approaches. In addition, the relatively restrained threshold in the present study may exclude certain proteins from the analysis, which leads to only a small number of proteins with significant differences between EC-Exo and SC-Exo. However, the excluded cargo proteins could potentially contribute to exosome function in recipient cells. It should be emphasized that additional experiments are warranted to investigate the effect of specific individual cargo proteins on neurovascular and myelin functions.

In summary, our proteomic analysis indicates that proteins involved in neurovascular function were abundant in the EC-Exo cargo, while SC-Exo cargo had more lipid metabolism proteins that regulate nerve myelination formation and function. The present study provides molecular insight into the therapeutic benefit of both SC-Exo and EC-Exo, and may lead to generation of exosomes whose protein content is engineered to enhance their therapeutic benefit for neuropathy.

## Supporting information

S1 Raw images (PDF)

S1 Table Antibodies used for Western blots. (DOCX)

S2 Table Protein expressed only in EC-Exo. (DOCX)

S3 Table Protein expressed only in SC-Exo. (DOCX)

S4 Table Abundant protein expressed in EC-Exo. (DOCX)

S5 Table Abundant protein expressed in SC-Exo. (DOCX)
