# Supplementary material for: Comparative proteomic analysis of exosomes derived from endothelial cells and Schwann cells
Source: PLoS One. 2023 Aug 18;18(8):e0290155. doi: 10.1371/journal.pone.0290155 (PMC10437921; doi:10.1371/journal.pone.0290155)
Supplement: S1 Raw images — (PDF) [file pone.0290155.s001.pdf]

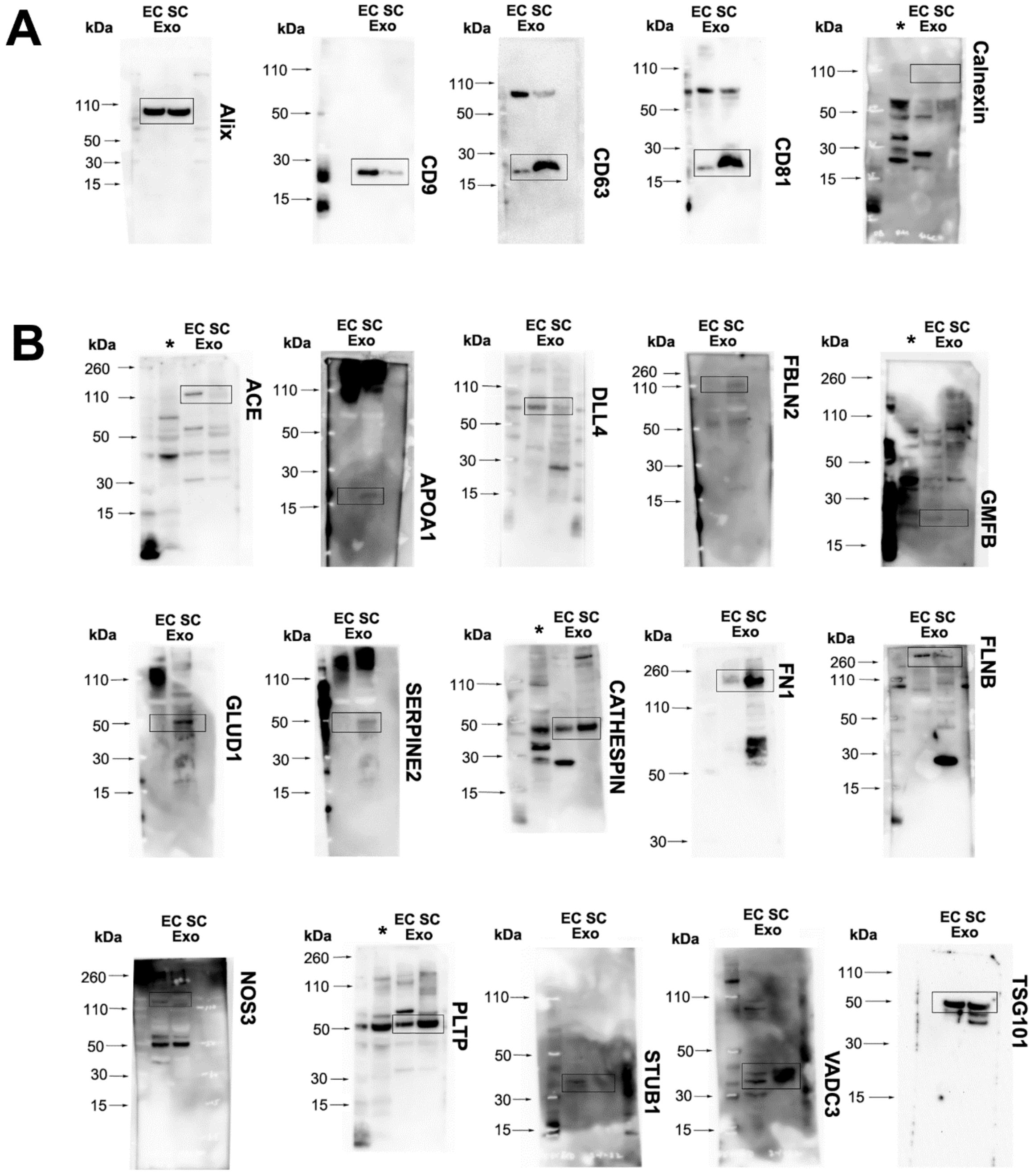

S1 Raw images. Raw data of Western blots. (A) Raw date of Western blots used in Figure 1C. (B) Raw data of Western blots used in Figure 7.

* Data in this column is not relevant to the present study.
