# Supplementary material for: Comparative proteomic analysis of exosomes derived from endothelial cells and Schwann cells
Source: PLoS One. 2023 Aug 18;18(8):e0290155. doi: 10.1371/journal.pone.0290155 (PMC10437921; doi:10.1371/journal.pone.0290155)
Supplement: S1 Table — (DOCX) [file pone.0290155.s002.docx]

**S1 Table. Antibodies used for Western Blots**

| Antibody name | Company and catalog number | concentration |
| --- | --- | --- |
| Anti-Alix | Cell Signaling, 2171 | 1:500 |
| Anti-CD9 | Biolegend, 699401 | 1:500 |
| Anti-CD63 | Santa Cruz, sc15363 | 1:500 |
| Anti-CD81 | Abcam, ab109201 | 1:500 |
| Anti-Calnexin | Abcam, ab223052 | 1:500 |
| Anti-ACE | Abclonal, A2805 | 1:500 |
| Anti-APOA1 | Abclonal, A4163 | 1:500 |
| Anti-CATHEPSIN | Abclonal, A19005 | 1:1000 |
| Anti-DLL4 | Abclonal, A12943 | 1:500 |
| Anti-FBLN2 | Abclonal, A16849 | 1:1000 |
| Anti-FN | Abclonal, A12977 | 1:500 |
| Anti-FLNB | Abclonal, A2481 | 1:500 |
| Anti-GLUD1 | Abclonal, A7631 | 1:500 |
| Anti-GMFB | Abclonal, A8652 | 1:500 |
| Anti-NOS3 | Santa Cruz, sc654 | 1:250 |
| Anti-PLTP | Abclonal, A5628 | 1:750 |
| Anti-SERPINE2 | Abclonal, A14540 | 1:750 |
| Anti-STUB1 | Abclonal, A11751 | 1:500 |
| Anti-VDAC3 | Abclonal, A11751 | 1:500 |
| Anti-TSG101 | Thermofisher, MA5-37764 | 1:500 |
| Anti-Rat IgG | Donkey 712-035-150 | 1:1000 |
| Anti-Mouse IgG | Donkey 715-035-150 | 1:1000 |
| Anti-Rabbit IgG | Donkey 711-035-152 | 1:1000 |
