# Supplementary material for: Comparative proteomic analysis of exosomes derived from endothelial cells and Schwann cells
Source: PLoS One. 2023 Aug 18;18(8):e0290155. doi: 10.1371/journal.pone.0290155 (PMC10437921; doi:10.1371/journal.pone.0290155)
Supplement: S2 Table — (DOCX) [file pone.0290155.s003.docx]

| S2 Table. Protein expressed only in EC-Exo | | | | | |
| --- | --- | --- | --- | --- | --- |
| Protein ID | | **Gene name** | | | **Protein name** |
| Q62181 | | | Sema3c | Semaphorin-3C | |
| P58022 | | | Loxl2 | Lysyl oxidase homolog 2 | |
| Q7TNP2 | | | Ppp2r1b | Serine/threonine-protein phosphatase 2A 65 kDa regulatory subunit A beta isoform | |
| P23242 | | | Gja1 | Gap junction alpha-1 protein | |
| Q07797 | | | Lgals3bp | Galectin-3-binding protein | |
| P21447 | | | Abcb1a | Multidrug resistance protein 1A | |
| Q9Z2X1 | | | Hnrnpf | Heterogeneous nuclear ribonucleoprotein F | |
| Q9WUA3 | | | Pfkp | ATP-dependent 6-phosphofructokinase, platelet type | |
| Q8CFG0 | | | Sulf2 | Extracellular sulfatase Sulf-2 | |
| O89001 | | | Cpd | Carboxypeptidase D | |
| Q8BZZ3 | | | Wwp1 | NEDD4-like E3 ubiquitin-protein ligase WWP1 | |
| P47740 | | | Aldh3a2 | Fatty aldehyde dehydrogenase | |
| P23249 | | | Mov10 | Putative helicase MOV-10 | |
| Q68FL6 | | | Mars | Methionine--tRNA ligase, cytoplasmic | |
| P22777 | | | Serpine1 | Plasminogen activator inhibitor 1 | |
| Q80W68 | | | Kirrel1 | Kin of IRRE-like protein 1 | |
| Q9QX15 | | | Clca3a1 | Calcium-activated chloride channel regulator 3A-1 | |
| P97427 | | | Crmp1 | Dihydropyrimidinase-related protein 1 | |
| P59108 | | | Cpne2 | Copine-2 | |
| O08808 | | | Diaph1 | Protein diaphanous homolog 1 | |
| Q9JJ28 | | | Flii | Protein flightless-1 homolog | |
| P01901 | | | H2-K1 | H-2 class I histocompatibility antigen, K-B alpha chain | |
| Q62318 | | | Trim28 | Transcription intermediary factor 1-beta | |
| Q99KH8 | | | Stk24 | Serine/threonine-protein kinase 24 | |
| P35285 | | | Rab22a | Ras-related protein Rab-22A | |
| Q3UU96 | | | Cdc42bpa | Serine/threonine-protein kinase MRCK alpha | |
| Q5FWK3 | | | Arhgap1 | Rho GTPase-activating protein 1 | |
| P29268 | | | Ctgf | Connective tissue growth factor | |
| Q62443 | | | Nptx1 | Neuronal pentraxin-1 | |
| Q62448 | | | Eif4g2 | Eukaryotic translation initiation factor 4 gamma 2 | |
| Q03137 | | | Epha4 | Ephrin type-A receptor 4 | |
| O35295 | | | Purb | Transcriptional activator protein Pur-beta | |
| Q9D9V3 | | | Echdc1 | Ethylmalonyl-CoA decarboxylase | |
| Q99JB8 | | | Pacsin3 | Protein kinase C and casein kinase II substrate protein 3 | |
| P48678 | | | Lmna | Prelamin-A/C | |
| P27808 | | | Mgat1 | Alpha-1,3-mannosyl-glycoprotein 2-beta-N-acetylglucosaminyltransferase | |
| D3YXG0 | | | Hmcn1 | Hemicentin-1 | |
| Q6PB93 | | | Galnt2 | Polypeptide N-acetylgalactosaminyltransferase 2 | |
| Q6PDQ2 | | | Chd4 | Chromodomain-helicase-DNA-binding protein 4 | |
| Q9WU60 | | | Atrn | Attractin | |
| Q811D0 | | | Dlg1 | Disks large homolog 1 | |
| Q8BHD7 | | | Ptbp3 | Polypyrimidine tract-binding protein 3 | |
| Q9EPK8 | | | Trpv4 | Transient receptor potential cation channel subfamily V member 4 | |
| Q8BML9 | | | Qars | Glutamine--tRNA ligase | |
| P81117 | | | Nucb2 | Nucleobindin-2 | |
| Q9CYL5 | | | Glipr2 | Golgi-associated plant pathogenesis-related protein 1 | |
| Q9Z1F9 | | | Uba2 | SUMO-activating enzyme subunit 2 | |
| P53811 | | | Pitpnb | Phosphatidylinositol transfer protein beta isoform | |
| P46664 | | | Adss | Adenylosuccinate synthetase isozyme 2 | |
| Q9JMH6 | | | Txnrd1 | Thioredoxin reductase 1, cytoplasmic | |
| Q80V26 | | | Impad1 | Inositol monophosphatase 3 | |
| Q9DAW9 | | | Cnn3 | Calponin-3 | |
| Q64008 | | | Rab34 | Ras-related protein Rab-34 | |
| Q3UMB9 | | | Washc4 | WASH complex subunit 4 | |
| Q99KK2 | | | Cmas | N-acylneuraminate cytidylyltransferase | |
| Q3U1V6 | | | Uevld | Ubiquitin-conjugating enzyme E2 variant 3 | |
| Q60805 | | | Mertk | Tyrosine-protein kinase Mer | |
| Q60751 | | | Igf1r | Insulin-like growth factor 1 receptor | |
| Q99JZ0 | | | Sdcbp2 | Syntenin-2 | |
| Q00609 | | | Cd80 | T-lymphocyte activation antigen CD80 | |
| Q5SWU9 | | | Acaca | Acetyl-CoA carboxylase 1 | |
| P97352 | | | S100a13 | Protein S100-A13 | |
| O35250 | | | Exoc7 | Exocyst complex component 7 | |
| Q99MN1 | | | Kars | Lysine--tRNA ligase | |
| P24547 | | | Impdh2 | Inosine-5'-monophosphate dehydrogenase 2 | |
| Q9WVA3 | | | Bub3 | Mitotic checkpoint protein BUB3 | |
| Q1HFZ0 | | | Nsun2 | tRNA (cytosine(34)-C(5))-methyltransferase | |
| Q60716 | | | P4ha2 | Prolyl 4-hydroxylase subunit alpha-2 | |
| Q8BHG1 | | | Nrdc | Nardilysin | |
| P49446 | | | Ptpre | Receptor-type tyrosine-protein phosphatase epsilon | |
| Q6X893 | | | Slc44a1 | Choline transporter-like protein 1 | |
| P17918 | | | Pcna | Proliferating cell nuclear antigen | |
| P61290 | | | Psme3 | Proteasome activator complex subunit 3 | |
| Q99M28 | | | Rnps1 | RNA-binding protein with serine-rich domain 1 | |
| Q61730 | | | Il1rap | Interleukin-1 receptor accessory protein | |
| Q61655 | | | Ddx19a | ATP-dependent RNA helicase DDX19A | |
| Q80VQ0 | | | Aldh3b1 | Aldehyde dehydrogenase family 3 member B1 | |
| P49717 | | | Mcm4 | DNA replication licensing factor MCM4 | |
| Q569Z5 | | | Ddx46 | Probable ATP-dependent RNA helicase DDX46 | |
| P70372 | | | Elavl1 | ELAV-like protein 1 | |
| Q6PIX5 | | | Rhbdf1 | Inactive rhomboid protein 1 | |
| Q8R550 | | | Sh3kbp1 | SH3 domain-containing kinase-binding protein 1 | |
| P49718 | | | Mcm5 | DNA replication licensing factor MCM5 | |
| Q8C2E7 | | | Washc5 | WASH complex subunit 5 | |
| Q91VU0 | | | Fam3c | Protein FAM3C | |
| O55135 | Eif6 | | | Eukaryotic translation initiation factor 6 | |
| Q3MI99 | Ccbe1 | | | Collagen and calcium-binding EGF domain-containing protein 1 | |
| P97310 | Mcm2 | | | DNA replication licensing factor MCM2 | |
| O88447 | Klc1 | | | Kinesin light chain 1 | |
| Q61468 | Msln | | | Mesothelin | |
| Q99MR6 | Srrt | | | Serrate RNA effector molecule homolog | |
| Q8BTH8 | Csnk1g1 | | | Casein kinase I isoform gamma-1 | |
| O70493 | Snx12 | | | Sorting nexin-12 | |
| P15116 | Cdh2 | | | Cadherin-2 | |
| Q99104 | Myo5a | | | Unconventional myosin-Va | |
| Q6A0A9 | FAM120A | | | Constitutive coactivator of PPAR-gamma-like protein 1 | |
| Q61881 | Mcm7 | | | DNA replication licensing factor MCM7 | |
| Q9JLZ8 | Sigirr | | | Single Ig IL-1-related receptor | |
| Q5Y5T1 | Zdhhc20 | | | Palmitoyltransferase ZDHHC20 | |
| Q91YT8 | Tmem63a | | | CSC1-like protein 1 | |
| Q9ES28 | Arhgef7 | | | Rho guanine nucleotide exchange factor 7 | |
| Q05909 | Ptprg | | | Receptor-type tyrosine-protein phosphatase gamma | |
| P53690 | Mmp14 | | | Matrix metalloproteinase-14 | |
| Q8BTY2 | Slc4a7 | | | Sodium bicarbonate cotransporter 3 | |
| Q6PFR5 | Tra2a | | | Transformer-2 protein homolog alpha | |
| O89086 | Rbm3 | | | RNA-binding protein 3 | |
| P83741 | Wnk1 | | | Serine/threonine-protein kinase WNK1 | |
| P70441 | Slc9a3r1 | | | Na(+)/H(+) exchange regulatory cofactor NHE-RF1 | |
| Q9DBU0 | Tm9sf1 | | | Transmembrane 9 superfamily member 1 | |
| Q9QY33 | Tspan3 | | | Tetraspanin-3 | |
| Q8CDN6 | Txnl1 | | | Thioredoxin-like protein 1 | |
| P62751 | Rpl23a | | | 60S ribosomal protein L23a | |
| P20444 | Prkca | | | Protein kinase C alpha type | |
| Q9JM61 | Thsd1 | | | Thrombospondin type-1 domain-containing protein 1 | |
| O35516 | Notch2 | | | Neurogenic locus notch homolog protein 2 | |
| Q61599 | Arhgdib | | | Rho GDP-dissociation inhibitor 2 | |
| Q9JI71 | Dll4 | | | Delta-like protein 4 | |
| Q8VBW1 | Slc6a8 | | | Sodium- and chloride-dependent creatine transporter 1 | |
| B0V2N1 | Ptprs | | | Receptor-type tyrosine-protein phosphatase S | |
| P49962 | Srp9 | | | Signal recognition particle 9 kDa protein | |
| P13439 | Umps | | | Uridine 5'-monophosphate synthase | |
| P25206 | Mcm3 | | | DNA replication licensing factor MCM3 | |
| P58242 | Smpdl3b | | | Acid sphingomyelinase-like phosphodiesterase 3b | |
| Q6PGC1 | Dhx29 | | | ATP-dependent RNA helicase DHX29 | |
| Q3THK7 | Gmps | | | GMP synthase [glutamine-hydrolyzing] | |
| Q8BWB6 | Steap2 | | | Metalloreductase STEAP2 | |
| P0DPB4 | Schip1 | | | Schwannomin-interacting protein 1 | |
| Q9CR16 | Ppid | | | Peptidyl-prolyl cis-trans isomerase D | |
| Q8VDZ4 | Zdhhc5 | | | Palmitoyltransferase ZDHHC5 | |
| P98195 | Atp9b | | | Probable phospholipid-transporting ATPase IIB | |
| Q6P9R2 | Oxsr1 | | | Serine/threonine-protein kinase | |
| Q8K019 | Bclaf1 | | | Bcl-2-associated transcription factor 1 | |
| Q60866 | Pter | | | Phosphotriesterase-related protein | |
| Q9CXW3 | Cacybp | | | Calcyclin-binding protein | |
| Q8BVY0 | Rsl1d1 | | | Ribosomal L1 domain-containing protein 1 | |
| P28740 | Kif2a | | | Kinesin-like protein KIF2A | |
| Q8CG48 | Smc2 | | | Structural maintenance of chromosomes protein 2 | |
| Q9CZU3 | Mtrex | | | Exosome RNA helicase MTR4 | |
| Q9CZ28 | Snf8 | | | Vacuolar-sorting protein SNF8 | |
| F8VQB6 | Myo10 | | | Unconventional myosin-X | |
| P83940 | Eloc | | | Elongin-C | |
| Q7TQJ1 | Marveld1 | | | MARVEL domain-containing protein 1 | |
| Q9JKC8 | Ap3m1 | | | AP-3 complex subunit mu-1 | |
| Q9JLI8 | Sart3 | | | Squamous cell carcinoma antigen recognized by T-cells 3 | |
| P82347 | Sgcd | | | Delta-sarcoglycan | |
| Q9DBG7 | Srpra | | | Signal recognition particle receptor subunit alpha | |
| P61759 | Vbp1 | | | Prefoldin subunit 3 | |
| P47856 | Gfpt1 | | | Glutamine--fructose-6-phosphate aminotransferase [isomerizing] 1 | |
| Q5XF89 | Atp13a3 | | | Probable cation-transporting ATPase 13A3 | |
| O70228 | Atp9a | | | Probable phospholipid-transporting ATPase IIA | |
| O89110 | Casp8 | | | Caspase-8 | |
| Q64455 | Ptprj | | | Receptor-type tyrosine-protein phosphatase eta | |
| O88848 | Arl6 | | | ADP-ribosylation factor-like protein 6 | |
| Q9D074 | Mgrn1 | | | E3 ubiquitin-protein ligase MGRN1 | |
| Q0VGY8 | Tanc1 | | | Protein TANC1 | |
| Q9ES46 | Parvb | | | Beta-parvin | |
| Q921C5 | Bicd2 | | | Protein bicaudal D homolog 2 | |
| Q3UHQ6 | Dop1b | | | Protein dopey-2 | |
| Q5PRF0 | Heatr5a | | | HEAT repeat-containing protein 5A | |
| Q01102 | Selp | | | P-selectin | |
| P31230 | Aimp1 | | | Aminoacyl tRNA synthase complex-interacting multifunctional protein 1 | |
| Q9QXA5 | Lsm4 | | | U6 snRNA-associated Sm-like protein LSm4 | |
| Q9WUL7 | Arl3 | | | ADP-ribosylation factor-like protein 3 | |
| Q9R1Z8 | Sorbs3 | | | Vinexin | |
| Q93092 | Taldo1 | | | Transaldolase | |
| Q8BMA6 | Srp68 | | | Signal recognition particle subunit SRP68 | |
| Q9CWX9 | Ddx47 | | | Probable ATP-dependent RNA helicase DDX47 | |
| P59708 | Sf3b6 | | | Splicing factor 3B subunit 6 | |
| P49769 | Psen1 | | | Presenilin-1 | |
| Q8VEN2 | Plet1 | | | Placenta-expressed transcript 1 protein | |
| P47931 | Fst | | | Follistatin | |
| P56546 | Ctbp2 | | | C-terminal-binding protein 2 | |
| A2AWA9 | Rabgap1 | | | Rab GTPase-activating protein 1 | |
| Q8R4G6 | Mgat5 | | | Alpha-1,6-mannosylglycoprotein 6-beta-N-acetylglucosaminyltransferase A | |
| Q9D2V7 | Coro7 | | | Coronin-7 | |
| Q6ZPE2 | Sbf1 | | | Myotubularin-related protein 5 | |
| Q9JKV1 | Adrm1 | | | Proteasomal ubiquitin receptor ADRM1 | |
| Q9R059 | Fhl3 | | | Four and a half LIM domains protein 3 | |
| Q9CX84 | Rgs19 | | | Regulator of G-protein signaling 19 | |
| Q3UMU9 | Hdgfl2 | | | Hepatoma-derived growth factor-related protein 2 | |
| Q8VD65 | Pik3r4 | | | Phosphoinositide 3-kinase regulatory subunit 4 | |
| Q9Z0P4 | Palm | | | Paralemmin-1 | |
| Q8VIK5 | Pear1 | | | Platelet endothelial aggregation receptor 1 | |
| Q921F4 | Hnrnpll | | | Heterogeneous nuclear ribonucleoprotein L-like | |
| P59325 | Eif5 | | | Eukaryotic translation initiation factor 5 | |
| P35505 | Fah | | | Fumarylacetoacetase | |
| P70193 | Lrig1 | | | Leucine-rich repeats and immunoglobulin-like domains protein 1 | |
| P37172 | Acvr1 | | | Activin receptor type-1 | |
| Q9Z1B3 | Plcb1 | | | 1-phosphatidylinositol 4,5-bisphosphate phosphodiesterase beta-1 | |
| A2AGT5 | Ckap5 | | | Cytoskeleton-associated protein 5 | |
| P16382 | Il4r | | | Interleukin-4 receptor subunit alpha | |
| Q8BGZ4 | Cdc23 | | | Cell division cycle protein 23 homolog | |
| Q8CCJ3 | Ufl1 | | | E3 UFM1-protein ligase 1 | |
| Q9Z0J1 | Reck | | | Reversion-inducing cysteine-rich protein with Kazal motifs | |
| P56389 | Cda | | | Cytidine deaminase | |
| Q61545 | Ewsr1 | | | RNA-binding protein EWS | |
| Q9R1P1 | Psmb3 | | | Proteasome subunit beta type-3 | |
| Q8K4Z5 | Sf3a1 | | | Splicing factor 3A subunit 1 | |
| P70290 | Mpp1 | | | 55 kDa erythrocyte membrane protein | |
| Q9CR00 | Psmd9 | | | 26S proteasome non-ATPase regulatory subunit 9 | |
| P52800 | Efnb2 | | | Ephrin-B2 | |
| Q91WQ3 | Yars | | | Tyrosine--tRNA ligase, cytoplasmic | |
| Q99PW4 | Tp53rk | | | EKC/KEOPS complex subunit Tp53rk | |
| P14576 | Srp54 | | | Signal recognition particle 54 kDa protein | |
| Q9JKK1 | Stx6 | | | Syntaxin-6 | |
| Q8VE98 | Cd276 | | | CD276 antigen | |
| P58871 | Tnks1bp1 | | | 182 kDa tankyrase-1-binding protein | |
| Q62433 | Ndrg1 | | | Protein NDRG1 | |
| Q9QUT0 | Rhag | | | Ammonium transporter Rh type A | |
| Q9R020 | Zranb2 | | | Zinc finger Ran-binding domain-containing protein 2 | |
| Q9QZC7 | Plekhb2 | | | Pleckstrin homology domain-containing family B member 2 | |
| Q64343 | Abcg1 | | | ATP-binding cassette sub-family G member 1 | |
| Q8K211 | Slc31a1 | | | High affinity copper uptake protein 1 | |
| Q8BRN9 | Cc2d1b | | | Coiled-coil and C2 domain-containing protein 1B | |
| A2RT67 | Dennd3 | | | DENN domain-containing protein 3 | |
| Q76KF0 | Sema6d | | | Semaphorin-6D | |
| P41233 | Abca1 | | | ATP-binding cassette sub-family A member 1 | |
| Q8R3B1 | Plcd1 | | | 1-phosphatidylinositol 4,5-bisphosphate phosphodiesterase delta-1 | |
| Q6ZQA6 | Igsf3 | | | Immunoglobulin superfamily member 3 | |
| Q64324 | Stxbp2 | | | Syntaxin-binding protein 2 | |
| Q9CWR7 | Steap1 | | | Metalloreductase STEAP1 | |
| D3YXK2 | Safb | | | Scaffold attachment factor B1 | |
| Q60875 | Arhgef2 | | | Rho guanine nucleotide exchange factor 2 | |
| Q9D771 | Tmem206 | | | Transmembrane protein 206 | |
| Q9CQ76 | Nepn | | | Nephrocan | |
| P49710 | Hcls1 | | | Hematopoietic lineage cell-specific protein | |
| Q9WV80 | Snx1 | | | Sorting nexin-1 | |
| P40224 | Cxcl12 | | | Stromal cell-derived factor 1 | |
| Q8BUK6 | Hook3 | | | Protein Hook homolog 3 | |
| Q9R1P3 | Psmb2 | | | Proteasome subunit beta type-2 | |
| Q8CIG8 | Prmt5 | | | Protein arginine N-methyltransferase 5 | |
| Q8C7K6 | Pcyox1l | | | Prenylcysteine oxidase-like | |
| Q6PAL8 | Dennd5a | | | DENN domain-containing protein 5A | |
| Q3TJD7 | Pdlim7 | | | PDZ and LIM domain protein 7 | |
| Q9DAW6 | Prpf4 | | | U4/U6 small nuclear ribonucleoprotein Prp4 | |
| Q501J7 | Phactr4 | | | Phosphatase and actin regulator 4 | |
| P54822 | Adsl | | | Adenylosuccinate lyase | |
| Q5SF07 | Igf2bp2 | | | Insulin-like growth factor 2 mRNA-binding protein 2 | |
| Q99LS3 | Psph | | | Phosphoserine phosphatase | |
| Q8K2Y9 | Ccm2 | | | Cerebral cavernous malformations protein 2 homolog | |
| Q3UPF5 | Zc3hav1 | | | Zinc finger CCCH-type antiviral protein 1 | |
| Q9CX34 | Sugt1 | | | Protein SGT1 homolog | |
| P24788 | Cdk11b | | | Cyclin-dependent kinase 11B | |
| Q9QUH0 | Glrx | | | Glutaredoxin-1 | |
| O89016 | Abcd4 | | | ATP-binding cassette sub-family D member 4 | |
| Q9EQ61 | Pes1 | | | Pescadillo homolog | |
| Q8BFQ4 | Wdr82 | | | WD repeat-containing protein 82 | |
| Q8CCB4 | Vps53 | | | Vacuolar protein sorting-associated protein 53 homolog | |
| Q60780 | Gas7 | | | Growth arrest-specific protein 7 | |
| Q8BL66 | Eea1 | | | Early endosome antigen 1 | |
| Q60767 | Ly75 | | | Lymphocyte antigen 75 | |
| Q6TYB5 | Fez2 | | | Fasciculation and elongation protein zeta-2 | |
| Q9D154 | Serpinb1a | | | Leukocyte elastase inhibitor A | |
| P28862 | Mmp3 | | | Stromelysin-1 | |
| P41241 | Csk | | | Tyrosine-protein kinase CSK | |
| Q6A051 | Atrnl1 | | | Attractin-like protein 1 | |
| P28659 | Celf1 | | | CUGBP Elav-like family member 1 | |
| Q3UM45 | Ppp1r7 | | | Protein phosphatase 1 regulatory subunit 7 | |
| Q8K1A6 | Cc2d1a | | | Coiled-coil and C2 domain-containing protein 1A | |
| Q9DCD5 | Tjap1 | | | Tight junction-associated protein 1 | |
| P11103 | Parp1 | | | Poly [ADP-ribose] polymerase 1 | |
| Q8VIM0 | Havcr2 | | | Hepatitis A virus cellular receptor 2 homolog | |
| Q02788 | Col6a2 | | | Collagen alpha-2(VI) chain | |
| Q8CJ53 | Trip10 | | | Cdc42-interacting protein 4 | |
| Q75N73 | Slc39a14 | | | Zinc transporter ZIP14 | |
| Q61333 | Tnfaip2 | | | Tumor necrosis factor alpha-induced protein 2 | |
| P23506 | Pcmt1 | | | Protein-L-isoaspartate(D-aspartate) O-methyltransferase | |
| Q9Z1K5 | Arih1 | | | E3 ubiquitin-protein ligase ARIH1 | |
| Q6ZQA0 | Nbeal2 | | | Neurobeachin-like protein 2 | |
| Q9R087 | Gpc6 | | | Glypican-6 | |
| Q8VDU5 | Snrk | | | SNF-related serine/threonine-protein kinase | |
| Q5SUF2 | Luc7l3 | | | Luc7-like protein 3 | |
| O70274 | Ptp4a2 | | | Protein tyrosine phosphatase type IVA 2 | |
| Q8BX35 | Eda2r | | | Tumor necrosis factor receptor superfamily member 27 | |
| Q8CIH5 | Plcg2 | | | 1-phosphatidylinositol 4,5-bisphosphate phosphodiesterase gamma-2 | |
| Q80U95 | Ube3c | | | Ubiquitin-protein ligase E3C | |
| Q8C033 | Arhgef10 | | | Rho guanine nucleotide exchange factor 10 | |
| P98156 | Vldlr | | | Very low-density lipoprotein receptor | |
| P32233 | Drg1 | | | Developmentally-regulated GTP-binding protein 1 | |
| Q8C351 | Layn | | | Layilin | |
| Q60673 | Ptprn | | | Receptor-type tyrosine-protein phosphatase-like N | |
| Q8C145 | Slc39a6 | | | Zinc transporter ZIP6 | |
| P54227 | Stmn1 | | | Stathmin | |
| Q8BNJ3 | Nmnat2 | | | Nicotinamide/nicotinic acid mononucleotide adenylyltransferase 2 | |
| Q08857 | Cd36 | | | Platelet glycoprotein 4 | |
| Q9DCD6 | Gabarap | | | Gamma-aminobutyric acid receptor-associated protein | |
| A2ASQ1 | Agrn | | | Agrin | |
| Q9R1S8 | Capn7 | | | Calpain-7 | |
| Q80U72 | Scrib | | | Protein scribble homolog | |
| P62080 | Tspan5 | | | Tetraspanin-5 | |
| Q9R0U0 | Srsf10 | | | Serine/arginine-rich splicing factor 10 | |
| Q61136 | Prpf4b | | | Serine/threonine-protein kinase PRP4 homolog | |
| Q8BGT6 | Micall1 | | | MICAL-like protein 1 | |
| Q8R5A3 | Apbb1ip | | | Amyloid beta A4 precursor protein-binding family B member 1-interacting protein | |
| Q9R0C8 | Vav3 | | | Guanine nucleotide exchange factor VAV3 | |
| Q6RHR9 | Magi1 | | | Membrane-associated guanylate kinase, WW and PDZ domain-containing protein 1 | |
| Q8CH18 | Ccar1 | | | Cell division cycle and apoptosis regulator protein 1 | |
| P56812 | Pdcd5 | | | Programmed cell death protein 5 | |
| Q9JKF6 | Nectin1 | | | Nectin-1 | |
| P51125 | Cast | | | Calpastatin | |
| Q8BTZ5 | Ankrd46 | | | Ankyrin repeat domain-containing protein 46 | |
| Q8BVF7 | Aph1a | | | Gamma-secretase subunit APH-1A | |
| Q8K2Y3 | Eva1b | | | Protein eva-1 homolog B | |
| Q9QXS6 | Dbn1 | | | Drebrin | |
| P48428 | Tbca | | | Tubulin-specific chaperone A | |
| Q8C525 | Mb21d2 | | | Protein MB21D2 | |
| Q7M759 | Abhd17b | | | Alpha/beta hydrolase domain-containing protein 17B | |
| Q5M8N0 | Cnrip1 | | | CB1 cannabinoid receptor-interacting protein 1 | |
| Q80Z96 | Vangl1 | | | Vang-like protein 1 | |
| Q4KMM3 | Oxr1 | | | Oxidation resistance protein 1 | |
| Q8CJ26 | Nradd | | | Death domain-containing membrane protein NRADD | |
