# Supplementary material for: Comparative proteomic analysis of exosomes derived from endothelial cells and Schwann cells
Source: PLoS One. 2023 Aug 18;18(8):e0290155. doi: 10.1371/journal.pone.0290155 (PMC10437921; doi:10.1371/journal.pone.0290155)
Supplement: S3 Table — (DOCX) [file pone.0290155.s004.docx]

| S3 Table. Protein expressed only in SC-Exo | | | | |  |  |
| --- | --- | --- | --- | --- | --- | --- |
| Protein ID | **Gene name** | **Protein name** |  | |  |  |
| Thbs1 | P35441 | Thrombospondin-1 |  | |  |  |
| Vcan | Q62059 | Versican core protein |  | |  |  |
| Col15a1 | O35206 | Collagen alpha-1(XV) chain | | |  |  |
| Krt6a | P50446 | Keratin, type II cytoskeletal 6A | | |  |  |
| Emilin1 | Q99K41 | EMILIN-1 |  | |  |  |
| Pcolce | Q61398 | Procollagen C-endopeptidase enhancer 1 | |  | | |
| Cryab | P23927 | Alpha-crystallin B chain |  | |  |  |
| Efemp2 | Q9WVJ9 | EGF-containing fibulin-like extracellular matrix protein 2 | | | | |
| Hspb1 | P14602 | Heat shock protein beta-1 | | |  |  |
| Glud1 | P26443 | Glutamate dehydrogenase 1, mitochondrial | |  | | |
| Gnao1 | P18872 | Guanine nucleotide-binding protein G(o) subunit alpha | | | | |
| Serpine2 | Q07235 | Glia-derived nexin |  | |  |  |
| Lcp1 | Q61233 | Plastin-2 |  | |  |  |
| Nid2 | O88322 | Nidogen-2 |  | |  |  |
| C1qtnf1 | Q9QXP7 | Complement C1q tumor necrosis factor-related protein 1 | | | | |
| Hspe1 | Q64433 | 10 kDa heat shock protein, mitochondrial | |  | | |
| Prkcsh | O08795 | Glucosidase 2 subunit beta | | |  |  |
| Loxl3 | Q9Z175 | Lysyl oxidase homolog 3 |  | |  |  |
| Naa25 | Q8BWZ3 | N-alpha-acetyltransferase 25, NatB auxiliary subunit | | | | |
| Sec61a1 | P61620 | Protein transport protein Sec61 subunit alpha isoform 1 | | | | |
| Aebp1 | Q640N1 | Adipocyte enhancer-binding protein 1 | |  | | |
| Phb2 | O35129 | Prohibitin-2 |  | |  |  |
| Adh7 | Q64437 | Alcohol dehydrogenase class 4 mu/sigma chain | | | | |
| Igfbp2 | P47877 | Insulin-like growth factor-binding protein 2 | |  | | |
| F13a1 | Q8BH61 | Coagulation factor XIII A chain | | |  |  |
| Txndc5 | Q91W90 | Thioredoxin domain-containing protein 5 | |  | | |
| Golim4 | Q8BXA1 | Golgi integral membrane protein 4 | | |  |  |
| Pgrmc2 | Q80UU9 | Membrane-associated progesterone receptor component 2 | | | | |
| Itga7 | Q61738 | Integrin alpha-7 |  | |  |  |
| Sec61b | Q9CQS8 | Protein transport protein Sec61 subunit beta | |  | | |
| Tmpo | Q61033 | Lamina-associated polypeptide 2, isoforms alpha/zeta | | | | |
| Adamtsl1 | Q8BLI0 | ADAMTS-like protein 1 |  | |  |  |
| Mtmr12 | Q80TA6 | Myotubularin-related protein 12 | | |  |  |
| Ssr1 | Q9CY50 | Translocon-associated protein subunit alpha | |  | | |
| Slc25a24 | Q8BMD8 | Calcium-binding mitochondrial carrier protein SCaMC-1 | | | | |
| Itpr3 | P70227 | Inositol 1,4,5-trisphosphate receptor type 3 | |  | | |
| Hsd17b4 | P51660 | Peroxisomal multifunctional enzyme type 2 | |  | | |
| Tg | O08710 | Thyroglobulin |  | |  |  |
| Fgg | Q8VCM7 | Fibrinogen gamma chain |  | |  |  |
| Mgst1 | Q91VS7 | Microsomal glutathione S-transferase 1 | |  | | |
| Tmem43 | Q9DBS1 | Transmembrane protein 43 | | |  |  |
| B4gat1 | Q8BWP8 | Beta-1,4-glucuronyltransferase 1 | | |  |  |
| Pef1 | Q8BFY6 | Peflin |  | |  |  |
| Tmem33 | Q9CR67 | Transmembrane protein 33 | | |  |  |
| Cmpk1 | Q9DBP5 | UMP-CMP kinase |  | |  |  |
| Rbm25 | B2RY56 | RNA-binding protein 25 |  | |  |  |
| Erp29 | P57759 | Endoplasmic reticulum resident protein 29 | |  | | |
| Cpt1a | P97742 | Carnitine O-palmitoyltransferase 1, liver isoform | | | | |
| Mlec | Q6ZQI3 | Malectin |  | |  |  |
| Gpd2 | Q64521 | Glycerol-3-phosphate dehydrogenase, mitochondrial | | | | |
| Col12a1 | Q60847 | Collagen alpha-1(XII) chain | | |  |  |
| Cpq | Q9WVJ3 | Carboxypeptidase Q |  | |  |  |
| Npc2 | Q9Z0J0 | NPC intracellular cholesterol transporter 2 | |  | | |
| Arfgap1 | Q9EPJ9 | ADP-ribosylation factor GTPase-activating protein 1 | | | | |
| Spcs2 | Q9CYN2 | Signal peptidase complex subunit 2 | | |  |  |
| Rela | Q04207 | Transcription factor p65 |  | |  |  |
| Etfb | Q9DCW4 | Electron transfer flavoprotein subunit beta | |  | | |
| Angptl3 | Q9R182 | Angiopoietin-related protein 3 | | |  |  |
| Nampt | Q99KQ4 | Nicotinamide phosphoribosyltransferase | |  | | |
| Fgb | Q8K0E8 | Fibrinogen beta chain |  | |  |  |
| Bpgm | P15327 | Bisphosphoglycerate mutase | | |  |  |
| Fuca1 | Q99LJ1 | Tissue alpha-L-fucosidase | | |  |  |
| Cds2 | Q99L43 | Phosphatidate cytidylyltransferase 2 | |  | | |
| Angptl2 | Q9R045 | Angiopoietin-related protein 2 | | |  |  |
| Flrt3 | Q8BGT1 | Leucine-rich repeat transmembrane protein FLRT3 | | | | |
| Itih5 | Q8BJD1 | Inter-alpha-trypsin inhibitor heavy chain H5 | |  | | |
| Mras | O08989 | Ras-related protein M-Ras | | |  |  |
| Pcdh12 | O55134 | Protocadherin-12 |  | |  |  |
| Entpd2 | O55026 | Ectonucleoside triphosphate diphosphohydrolase 2 | | | | |
| Sparcl1 | P70663 | SPARC-like protein 1 |  | |  |  |
| Atxn3 | Q9CVD2 | Ataxin-3 |  | |  |  |
| Aldh1a7 | O35945 | Aldehyde dehydrogenase, cytosolic 1 | |  | | |
| Cyb5a | P56395 | Cytochrome b5 |  | |  |  |
