# Supplementary material for: Comparative proteomic analysis of exosomes derived from endothelial cells and Schwann cells
Source: PLoS One. 2023 Aug 18;18(8):e0290155. doi: 10.1371/journal.pone.0290155 (PMC10437921; doi:10.1371/journal.pone.0290155)
Supplement: S4 Table — (DOCX) [file pone.0290155.s005.docx]

**S4 Table. Abundant protein expressed in EC-Exo**

| Protein ID | Gene name | Protein name | P value | Fold Change |
| --- | --- | --- | --- | --- |
| P62331 | Arf6 | ADP-ribosylation factor 6 | 0.0044 | 2.50 |
| O09131 | Gsto1 | Glutathione S-transferase omega-1 | 0.0069 | 2.00 |
| P08030 | Aprt | Adenine phosphoribosyltransferase | 0.0077 | 6.33 |
| Q61990 | Pcbp2 | Poly(rC)-binding protein 2 | 0.0082 | 4.67 |
| Q9Z127 | Slc7a5 | Large neutral amino acids transporter small subunit 1 | 0.0082 | 3.75 |
| Q99K85 | Psat1 | Phosphoserine aminotransferase | 0.0093 | 8.67 |
| P02469 | Lamb1 | Laminin subunit beta-1 | 0.0108 | 5.69 |
| Q9JLQ0 | Cd2ap | CD2-associated protein | 0.0121 | 10.00 |
| Q9CZX8 | Rps19 | 40S ribosomal protein S19 | 0.0121 | 5.50 |
| P50543 | S100a11 | Protein S100-A11 | 0.0121 | 2.80 |
| P41731 | Cd63 | CD63 antigen | 0.0121 | 2.00 |
| Q62188 | Dpysl3 | Dihydropyrimidinase-related protein 3 | 0.0136 | 3.40 |
| P63242 | Eif5a | Eukaryotic translation initiation factor 5A-1 | 0.0161 | 4.67 |
| O08599 | Stxbp1 | Syntaxin-binding protein 1 | 0.0161 | 2.83 |
| P63037 | Dnaja1 | DnaJ homolog subfamily A member 1 | 0.0166 | 5.60 |
| Q9DBJ1 | Pgam1 | Phosphoglycerate mutase 1 | 0.0194 | 4.33 |
| Q9QUR6 | Prep | Prolyl endopeptidase | 0.0194 | 4.33 |
| P04925 | Prnp | Major prion protein | 0.0194 | 4.33 |
| Q9JJR8 | Tmem9b | Transmembrane protein 9B | 0.0198 | 8.00 |
| Q9WV95 | Phlda3 | Pleckstrin homology-like domain family A member 3 | 0.0198 | 3.33 |
| Q924C6 | Loxl4 | Lysyl oxidase homolog 4 | 0.0215 | 7.33 |
| P68181 | Prkacb | cAMP-dependent protein kinase catalytic subunit beta | 0.0215 | 8.50 |
| Q8BHL4 | Gprc5a | Retinoic acid-induced protein 3 | 0.0241 | 3.86 |
| Q6A028 | Swap70 | Switch-associated protein 70 | 0.0283 | 14.00 |
| Q9ER72 | Cars | Cysteine--tRNA ligase, cytoplasmic | 0.0299 | 9.00 |
| P28656 | Nap1l1 | Nucleosome assembly protein 1-like 1 | 0.0299 | 3.67 |
| Q91V12 | Acot7 | Cytosolic acyl coenzyme A thioester hydrolase | 0.0299 | 3.67 |
| Q8BKC5 | Ipo5 | Importin-5 | 0.0299 | 2.60 |
| P70398 | Usp9x | Probable ubiquitin carboxyl-terminal hydrolase FAF-X | 0.0365 | 4.50 |
| O08602 | Raet1a | Retinoic acid early-inducible protein 1-alpha | 0.0378 | 6.00 |
| Q8CFI0 | Nedd4l | E3 ubiquitin-protein ligase NEDD4-like | 0.0378 | 6.00 |
| Q8BFW7 | Lpp | Lipoma-preferred partner homolog | 0.0378 | 6.00 |
| Q11011 | Npepps | Puromycin-sensitive aminopeptidase | 0.0378 | 3.50 |
| P45377 | Akr1b8 | Aldose reductase-related protein 2 | 0.0378 | 3.50 |
| P56485 | Ackr3 | Atypical chemokine receptor 3 | 0.0378 | 2.67 |
| P70349 | Hint1 | Histidine triad nucleotide-binding protein 1 | 0.0378 | 2.25 |
| Q9WTQ5 | Akap12 | A-kinase anchor protein 12 | 0.0378 | 2.00 |
| Q9CQ65 | Mtap | S-methyl-5'-thioadenosine phosphorylase | 0.0385 | 8.00 |
| P02468 | Lamc1 | Laminin subunit gamma-1 | 0.0385 | 3.31 |
| Q9WUD1 | Stub1 | STIP1 homology and U box-containing protein 1 | 0.0389 | 12.00 |
| P14152 | Mdh1 | Malate dehydrogenase, cytoplasmic | 0.0389 | 2.83 |
| Q61001 | Lama5 | Laminin subunit alpha-5 | 0.0396 | 2.78 |
| Q9JIZ9 | Plscr3 | Phospholipid scramblase 3 | 0.0422 | 3.86 |
| Q80X90 | Flnb | Filamin-B | 0.0438 | 3.07 |
| Q61072 | Adam9 | Disintegrin and metalloproteinase domain-containing protein 9 | 0.0440 | 7.33 |
| P12815 | Pdcd6 | Programmed cell death protein 6 | 0.0440 | 2.46 |
| Q8R4Y4 | Stab1 | Stabilin-1 | 0.0446 | 3.96 |
