# Supplementary material for: Comparative proteomic analysis of exosomes derived from endothelial cells and Schwann cells
Source: PLoS One. 2023 Aug 18;18(8):e0290155. doi: 10.1371/journal.pone.0290155 (PMC10437921; doi:10.1371/journal.pone.0290155)
Supplement: S5 Table — (DOCX) [file pone.0290155.s006.docx]

**S5 Table: Abundant protein expressed in SC-Exo**

| Protein ID | Gene name | Protein name | P value | Fold Change |
| --- | --- | --- | --- | --- |
| Q01149 | Col1a2 | Collagen alpha-2(I) chain | 0.0052 | 13.00 |
| Q60931 | Vdac3 | Voltage-dependent anion-selective channel protein 3 | 0.0136 | 3.78 |
| P11276 | Fn1 | Fibronectin | 0.0246 | 9.00 |
| Q00623 | Apoa1 | Apolipoprotein A-I | 0.0283 | 7.68 |
| Q64133 | Maoa | Amine oxidase [flavin-containing] A | 0.0299 | 2.67 |
| P37889 | Fbln2 | Fibulin-2 | 0.0315 | 3.50 |
| P83882 | Rpl36a | 60S ribosomal protein L36a | 0.0378 | 3.53 |
| Q07113 | Igf2r | Cation-independent mannose-6-phosphate receptor | 0.0465 | 2.18 |
